# Parallel operated hybrid Arithmetic-Salp swarm optimizer for optimal allocation of multiple distributed generation units in distribution networks

**DOI:** 10.1371/journal.pone.0264958

**Published:** 2022-04-13

**Authors:** Zeeshan Memon Anjum, Dalila Mat Said, Mohammad Yusri Hassan, Zohaib Hussain Leghari, Gul Sahar

**Affiliations:** 1 Centre of Electrical Energy Systems (CEES), Institute of Future Energy (IFE), Universiti Teknologi Malaysia (UTM), Johor Bahru, Johor, Malaysia; 2 School of Electrical Engineering (SKE), Faculty of Engineering, Universiti Teknologi Malaysia (UTM), Johor Bahru, Johor, Malaysia; 3 Department of Electrical Engineering, Mehran University of Engineering and Technology (MUET), SZAB Campus, Khairpur Mirs, Sindh, Pakistan; 4 Department of Electrical Engineering, Mehran University of Engineering and Technology (MUET), Jamshoro, Sindh, Pakistan; 5 School of Computing, Faculty of Engineering, Universiti Teknologi Malaysia (UTM), Johor Bahru, Johor, Malaysia; 6 Department of Computer Sciences, Karakoram International University, Gilgit-Baltistan, Pakistan; Universidad de Guadalajara, MEXICO

## Abstract

The installation of Distributed Generation (DG) units in the Radial Distribution Networks (RDNs) has significant potential to minimize active power losses in distribution networks. However, inaccurate size(s) and location(s) of DG units increase power losses and associated Annual Financial Losses (AFL). A comprehensive review of the literature reveals that existing analytical, metaheuristic and hybrid algorithms employed on DG allocation problems trap in local or global optima resulting in higher power losses. To address these limitations, this article develops a parallel hybrid Arithmetic Optimization Algorithm and Salp Swarm Algorithm (AOASSA) for the optimal sizing and placement of DGs in the RDNs. The proposed parallel hybrid AOASSA enables the mutual benefit of both algorithms, i.e., the exploration capability of the SSA and the exploitation capability of the AOA. The performance of the proposed algorithm has been analyzed against the hybrid Arithmetic Optimization Algorithm Particle Swarm Optimization (AOAPSO), Salp Swarm Algorithm Particle Swarm Optimization (SSAPSO), standard AOA, SSA, and Particle Swarm Optimization (PSO) algorithms. The results obtained reveals that the proposed algorithm produces quality solutions and minimum power losses in RDNs. The Power Loss Reduction (PLR) obtained with the proposed algorithm has also been validated against recent analytical, metaheuristic and hybrid optimization algorithms with the help of three cases based on the number of DG units allocated. Using the proposed algorithm, the PLR and associated AFL reduction of the 33-bus and 69-bus RDNs improved to 65.51% and 69.14%, respectively. This study will help the local distribution companies to minimize power losses and associated AFL in the long-term planning paradigm.

## Introduction

The power demand has witnessed stupendous growth with the rise of population. As a result, the amount of electricity generated must be raised to satisfy consumer demand, which has a significant impact on the economic development of countries. In this regard, DGs at local level sounds to be a promising solution. A Distributed Generator (DG) is a small electricity-generating unit that plays an essential role in improving the power market because of its compact size, high efficiency, low operating costs, and safety [1]. The DGs are smaller in size, ranging from a few kilowatts to 100 Megawatts [2]. In addition, DGs are directly integrated to the consumer side, unlike the centrally located power generating units situated far away from load centers and resulting in higher transmission losses [3–6]. However, connecting DG of inappropriate size to non-optimal location results in higher power losses and total cost, therefore offsetting the primary goal of connecting it to the distribution networks [7–10]. The optimal allocation of DGs, involves determining the best sizes and locations to satisfy the required goals while adhering to distribution network constraints. The allocation of DG units in distribution networks is a complex, nonlinear and combinatorial mixed-integer optimization problem since it incorporates both discrete (DG sizes) and continuous (DG locations) variables [11]. Therefore, the optimally sized and located DG units significantly maximize PLR in distribution networks [12–15]. The copper losses are gaining importance in the distribution network due to higher resistance to reactance ratio [16,17]. Copper loss (i.e., active power loss) in the distribution networks is estimated to dissipate roughly 13% of total produced power [18,19]. Therefore, the proposed study minimizes active power losses, instead of optimizing the total loss function. Several algorithms have been proposed in the literature to optimize DG size and location for maximizing PLR. However, with recent advances in metaheuristic algorithms, possible modifications in algorithms and various combinations among the recently introduced hybrid algorithms significantly improve the performance of the system.

The optimization techniques are broadly categorized into conventional (analytical) techniques, Meta-Heuristic Techniques (MHT), and hybrid techniques [20–22]. These techniques have a variety of applications in scheduling/commitment [23] of resources in short term planning or allocation of resources in long term planning [24]. The scope of the proposed study is limited to the long-term allocation of resources and these optimization techniques are targeted for resource allocation purpose. The optimal allocation of resources in the network help in minimizing the requirements for the system, power losses and cost. In real world problems, the optimal allocation of sensors in wireless sensor networks (WSNs) or Flexible AC Transmission Systems (FACTS) controllers in electrical transmission networks [25,26] have a profound impact on requirements of the system and network performance [27]. Like allocation of sensors or FACTS controllers, the optimal allocation of DG has a great impact on network power losses. A variety of research work has been highlighted in the literature on the optimal allocation of DG to solve power loss minimization problem with the help of analytical techniques. Rau and Wan [28] presented a second-order algorithm to obtain the optimal sizes and locations of DGs. Loss Sensitivity Factor (LSF) [29] was proposed for power loss reduction in 33-bus RDN. The results showed that the power losses were reduced by 30.48%, 52.32%, and 59.72%, with optimal allocation of one, two, and three DG units, respectively. Acharya et al. [30] developed the exact loss formula based on analytical expressions to obtain the optimal DG sizes and location for minimizing power losses. The results revealed that the power loss reduced by 47.32% and 62.86% in 33-bus and 69-bus networks, respectively. In [31], the optimal allocation of a single DG unit in the 69-bus RDN was carried out using an analytical technique. The results showed that with the optimal sizing and placement of one DG unit, power losses were reduced by 62.95%. A sensitivity analysis was performed by Ramesh et al. [32] for optimal DG allocation to minimize the power loss in the Tamil Nadu Electricity board 11kV distribution feeder and IEEE-37 bus distribution network. Though various analytical approaches were found from the existing literature, yet analytical techniques have less robustness, are based on a set of rules, linearization, and simplified assumptions, which makes them inappropriate for optimal allocation of DG units [33].

Meta-heuristic techniques (MHTs) have widely been adopted in the recent past to solve mixed-integer DG allocation problems. The wide adoption of MHTs became viable since they are flexible and follow gradient-free mechanisms. Therefore, the need to calculate the search space is eliminated, making them flexible in solving a wide range of optimization problems. Furthermore, MHTs lies in stochastic optimization techniques, therefore they take the benefit of random operators. These operators help MHTs to jump out of the local optima when solving real-world applications, which usually have many local solutions. Recently, various studies have witnessed the superiority of MHTs for optimal DG allocation problems. Teaching Learning-Based Optimization (TLBO) was employed by Sultana and Roy [34] to optimize the allocation of three DG units in the 33-bus and 69-bus distribution networks. It was observed that with the optimal allocation of three DGs, the losses were reduced by 64.20% in 33-bus RDN and by 67.82% in 69-bus RDN. Furthermore, in the same study, it was observed that Quasi Oppositional TLBO (QOTLBO) [34] performed better than TLBO producing loss reduction of 64.88% and 68.16% on 33-bus and 69-bus, respectively. The Tabu search (TS) method could solve simple problems but was limited in ability to address complex real-world application problems and was reliant on starting solutions. As a result, it was unsuitable for large-scale distribution network planning because finding a good starting solution was challenging. There was no guarantee of attaining a global optimum when employing TS for optimization. In another study [35], Krill Herd Algorithm (KHA) was used to optimize the allocation of three DG units in 33-bus and 69-bus test networks. The results revealed that a loss reduction of 64.26% and 69.09% were achieved in 33-bus and 69-bus test networks, respectively. The KHA was simple in concept, easy to implement, and could significantly exploit at the end of the search process but lacked a good search space in the exploration phase. Fuzzy AIS (Artificial Immune system) was implemented by Lalitha et al. [36] to minimize the power losses in the 33-bus RDN for optimal allocation of one, two, and three DG units. It was observed that the losses reduced by 37.71%, 42.43%, and 42.45% with the optimal allocation of one, two, and three DG units, respectively. However, the study did not consider voltage constraints; therefore, the global solution violated power system constraints. In addition, Artificial Immune System (AIS) is more intelligent than GA, thanks to guided mutation and duplication operators. However, tuning the mutation and duplication rates was a challenge for AIS application. In general, meta-heuristic optimization algorithms can resolve multi-dimensional problems as they are robust in nature; however, still deficit in terms of premature convergence or local optima stagnation. Despite of better performance than that of analytical approaches, MHTs were found to be comparatively less potential than hybrid approaches while dealing with combinatorial optimal DG allocation problem.

MHTs had a variety of challenges while dealing with DG allocation problems, for instance, premature convergence and slow response. These weaknesses were correlated with solution diversity in the search space. A trade-off between convergence rate and accuracy was ideal; however, the results were often unpredictable in complex real-world problems. One of the factors for premature convergence was the lack of solution diversity. Appropriate Solution diversity should be maintained in the search process to avoid local optima stagnation in the optimization algorithm. The hybridization of the two algorithms maintained solution diversity during the entire search process. The hybrid algorithms were widely adopted in modern applications, including recognition of COVID-19 disease from x-ray images [37], scheduling of nurses for COVID-19 patients [38], wind speed forecasting [39], digital currency forecasting [40], detection of solder paste defects [41], network security [42] etc. The enhanced balance between exploration and exploitation (in the search space) with the hybridization of two meta-heuristic algorithms had resulted in superior quality solutions in complex optimization problems. Hybrid algorithms combined the superiority of one algorithm with another. In other words, the limitations of one algorithm were significantly improved with the hybridization of other algorithms [43]. The Hybrid MHTs were more robust and produced quality solutions as compared to standard MHTs [33]. In the last decade, hybrid algorithms had attended great interest in DG allocation problems. Genetic Algorithm (GA) was hybridized with other techniques such as Monte Carlo [44], Hong’s two-point estimation technique [45], interior point approach [46], and the local search [47]. In [48], Particle Swarm Optimization (PSO) was used to locate dispersed sources, and a derivative-based analytical technique was utilized to compute the amount of injected power to minimize power losses. In [49], a recently proposed metaheuristic Symbiotic Organism Search (SOS) was combined with an analytical approach called zero bus load flow. Gandomkar et al. [50] proposed a new approach for efficient DG allocation in the distribution networks using a combination of GA and Simulated Annealing (SA) algorithms. In [51], a combination of analytical and metaheuristic, Loss Sensitivity Factor Simulated Annealing (LSFSA), was presented for optimal allocation of three DG units in 33- and 69-bus RDN. The results revealed that the power losses were reduced by 61.11% in 33-bus RDN and 67.95% in 69-bus RDN. To allocate single, double, and triple DG units in 33-bus and 69-bus RDNs, a hybridization of analytical and meta-heuristic PSO methods was used in [48]. The results revealed that the power loss reductions of 47.20%, 58.49%, and 65.35% were achieved with optimal allocation of 1, 2, and 3 DG units in 33-bus RDN. Furthermore, the copper losses were reduced by 62.95%, 68.09%, and 69.09%, with optimal allocation of one, two, and three DG units in 69-bus RDN. In another study [52], a hybrid Genetic algorithm-Particle swarm optimization (GA-PSO) technique was proposed for optimal allocation of three DG units in 33-bus and 69-bus RDNs. It was observed that the power losses reduced by 51.02% and 62.40% in 33- and 69-bus RDNs, respectively. Jamian et al. [53] implemented Rank Evolutionary Particle Swarm Optimization (RESPO) to minimize the power loss with optimal allocation of dual and triplet DG units in 33-bus RDN, which produced the power loss reductions of 44.68% and 63.55%, respectively. Genetic Algorithm and Intelligent Water Drop (GAIWD) [54] was implemented for optimal allocation of 3 DG units on 33-bus and 69-bus RDN. The results revealed that the power losses reduced by 47.63% and 64.04% on 33-bus and 69-bus systems, respectively. Hybrid Harmony Search Algorithm and Particle Artificial Bee Colony Algorithm (HSA-PABC) presented in [55] optimally allocated three DG units and produced the PLR of 65.49% and 68.12% for 33-bus and 69-bus RDNs, respectively. Recently, a combination of Tabu Search (TS) and Chu Beasley Genetic Algorithm (CBGA) was proposed for the optimal allocation of DG units [56]. The CBGA was utilized to size the DG units while the TS determined their placement. Lin et al. [57] proposed a hybrid technique to reduce real power loss, which combines an analytical LSF method for sizing DGs with a meta-heuristic and PSO method for sitting DGs based on optimal reactive power dispatch. In [58], the master stage used the Population-Based Incremental Learning (PPBIL) to optimize the location of the generators and the slave stage used Particle swarm optimization (PSO) to optimize the corresponding sizes. In [21], the Salp Swarm Algorithm (SSA) optimized the sites, while the analytical approach optimized the sizes of DGs. However, the problem occurred in dedicating one algorithm for optimal sizing and the other for optimal siting resulting in inaccurate size or site due to the limitation of an individual algorithm. A hybrid approach for loss reduction and voltage improvement was proposed in [59]; the position of DG was determined using an empirical discrete metaheuristic (EDM), while the size was determined using the steepest descent method (SD). However, in the later technique (SD), calculating exact step size was time consuming and challenging task, while non optimal selection of step size results in higher power losses. In [43], LSF was utilized to select candidate buses for DGs placements, while an analytical approach was used to find the optimal DG sizes for the combinations of candidate buses; these values were then used as the beginning values in the Sine cosine algorithm (SCA) to specify the DGs sizes. However, the final solutions of SCA were purely dependent on the LSF and analytical algorithm as both the algorithms decide the initial search space. The initial search of SCA was limited to the solutions of LSF and analytical algorithm which was not a practical approach. The contributions of recently developed hybrid techniques and the respective limitations applied on DG optimal DG allocation problem are presented in Table 1.

**Table 1:** Contributions and limitations of recently developed hybrid techniques for DG allocation.

Ref	Authors	Year	Contributions	limitations
[21]	A. Mohamed et.al.	2021	minimized power losses with hybrid analytical and SCA	Both algorithms were not utilized simultaneously for optimal sizing and location which produced local/global optima stagnation
[43]	Ali Selim et. al.	2021	Minimized Active power losses with LSF and analytical technique fed to SCA	Analytical and LSF technique feeding SCA would limit the initial search of SCA leading to sub-optimal size and locations of DGs
[60]	Ayman Awad et.al.	2021	Solved weighted sum multi-objective model with tunicate swarm algorithm/sine-cosine algorithm (TSA/SCA)	Though the improved TSA/SCA resulted in good exploration yet it lacked exploitation capabilities resulting in sub-optimal solution of DG sizes and locations
[59]	Francisco Carlos Rodrigues Coelho et al	2020	Hybridized EDM and SD to minimize power losses and introduced penalty factor to voltages at desired levels	Both algorithms were not utilized simultaneously for optimal sizing and locations. Furthermore, it was difficult to find appropriate step size in SD resulting in non-optimal sizing of DGs.
[58]	Luis Fernando Grisales-Noreña et.al.	2020	Proposed Master-slave combination of PPBIL and PSO to minimize the power losses	The individual algorithm did not search the optimal size and locations of DGs simultaneously. The mechanism would result in sub optimal solutions of DG sizes and locations.

The contemporary literature suggests that the hybrid MHTs have widely been adopted in the last decade. Hybrid algorithms allow the positive aspects of different algorithms and eliminate the inherited deficiencies present in standard metaheuristic algorithms. Even though a variety of work has been presented for minimizing losses with optimal allocation of DG units using various MHT taxonomies, there are numerous methodological limitations in it. One of the limitations observed is the dependency of the later algorithm on the former algorithm, which minimizes the search space of the later algorithm and traps it in local optima. The second limitation is that the dedication of one algorithm for optimal sizing and the other for optimal siting will result in inaccurate size or site due to the limitation of an individual algorithm. To address these limitations, it is imperative to develop a hybrid algorithm that can independently search the sizes and locations of DG units and is independent of initial search space. In this context, parallel hybrid AOASSA methodology has been developed, which provides an independent initial search space. The proposed hybrid algorithm runs independently to determine the optimal sizes and location of DG units. The associated power loss value is compared in every iteration, and the control variables (DG sizes and locations) of the weak algorithms are replaced with dominant algorithms. The overall combination of hybrid AOASSA avoids local optima stagnation and navigates towards the optimal solution. The parallel operated hybrid AOASSA taxonomy motivates the authors because of two reasons; firstly, the AOASSA mitigates the exploration limitations of AOA and exploitation limitations of SSA by utilizing the iterative parameters *c*_*1*_ (corresponding to SSA) and Math Operator Probability (MOP corresponding to AOA) in the exploration and exploitation phase, respectively. Secondly, the parallel operated AOASSA provides equal opportunity to AOA and SSA to search optimal sizes and locations individually. However, in the literature, it has been observed that one algorithm is dedicated to search optimal size while other algorithm searches the locations. Therefore, any unoptimized selection of size or location using a particular algorithm result in higher power losses. In addition, the parallel operated mechanism mitigates the probability of local optima stagnation. The proposed AOASSA runs two algorithms (AOA and SSA) in parallel throughout the entire search process, mitigating the uncertainty of poor exploration or exploitation.

In contrast to the highlighted research gaps and significance of the proposed algorithm, the main contributions of the proposed study are stated as below:

1. To develop a methodology based on parallel operated hybrid AOASSA.
2. To apply the developed parallel operated AOASSA optimizer for determining the optimal sizes and locations of multiple DG units in the radial distribution network for minimizing active power losses and associated Annual Financial Losses (AFL).
3. To analyze the performance of proposed parallel operated AOASSA on optimal DG allocation problem against standard version of AOA, SSA, PSO and their possible hybrid combinations (i.e., AOAPSO and SSAPSO) in terms of convergence quality, convergence speed, robustness, and annual financial losses.
4. To validate the performance of proposed parallel operated AOASSA in terms of PLR against recent analytical, metaheuristic and hybrid metaheuristic algorithms using standard 33-bus and 69-bus RDN.

This paper is organized as follows: First, the problem formulation and constraints are discussed. Second, the description of the proposed AOASSA adopted for the DG allocation problem is elaborated in detail. Third, results and discussions highlighting the performance of the proposed algorithm are discussed. In the end, the conclusions and future road maps based on the current study are summarized.

## Problem formulation

Since the resistance to reactance ratio is comparatively higher in distribution networks compared to transmission networks, therefore the resistive losses dominate the reactive power losses in distribution networks. It is estimated that 13% of losses are dissipated as copper losses in distribution networks. Therefore, the primary objective function of the proposed study is to minimize the active power losses. In addition, the power losses are translated in terms of AFL. The operational and topological constraints are considered while optimally sizing and sitting DG units

### Power Loss Reduction (PLR)

The dominance of active power losses in the distribution network makes active power losses more prevalent than reactive power losses. The system power loss is the aggregated sum of the active power loss across the individual branch and is generally stated in Eq (1).


$$Objective\;Function=Minimize(P_{loss})=Minimize\sum_{i=1}^{N}I_{i}^{2}R_{i}$$ (1)

Where *I*_*i*_ is the current flowing through the *i*^*th*^ branch, *R*_*i*_ is the resistance of the *i*^*th*^ branch and *N* represents number of buses. It must be highlighted that the power losses in branches depend upon the flows of currents in the respective branch, which is ultimately influenced by the power injections on the nodes connected to lines. Therefore, it is imperative to optimize the size and location of DGs such that minimum power losses are achieved. The term power loss reduction (%PLR) is expressed mathematically by Eq (2).


$$\%PLR=\frac{P_{loss(without\;DG)}-P_{loss(with\;DG)}}{P_{loss(without\;DG)}}\times 100$$ (2)

Where *P*_*loss*(*without DG*)_ and *P*_*loss*(*with DG*)_ are the power losses in the network without and with DG allocation, respectively. The main objective of the proposed study is to minimize active power losses in the network by optimally allocating the DGs using the proposed AOASSA. The reduction in power losses is translated in terms of economic losses. The installation of DGs reduces the energy intake from the main grid. However, non-optimal allocation of DGs increases power losses in the network, hence the financial losses also increase in the distribution network. The economic losses, translated in terms of energy losses, are defined by the term AFL and are given by Eq (3).


$$AFL=\frac{\$}{kwh}*P_{\left(loss\right)}*8760h$$ (3)

Where $/*kWh* is the per-unit cost of electricity and is taken as 0.052$ (0.2121MYR) [61], *P*_(*loss*)_ is the cumulative power loss in all branches and 8760 is the number of hours in 1 year.

### Distribution network constraints

The constraints are the set of boundary conditions that must be copped before obtaining the resultant solution. In terms of the distribution network, active power flow, reactive power flow, and bus voltage (p.u) are considered in Eqs (4–6), respectively.


$$\overset{Nbus}{\underset{j=1}{\sum }}P_{load}^{j}+\overset{N\;branches}{\underset{i=1}{\sum }}P_{loss}^{i}>\overset{Z\;DGs}{\underset{i=1}{\sum }}P_{DG}^{Z}$$ (4)

$$\overset{Nbus}{\underset{i=1}{\sum }}Q_{load}^{j}+\overset{N\;branches}{\underset{i=1}{\sum }}Q_{loss}^{i}>\overset{Z\;DGs}{\underset{i=1}{\sum }}Q_{DG}^{Z}$$ (5)

$$1.05\;p.u<V_{J}>0.95\;p.u$$ (6)

The active and reactive loads on the *j*^*th*^ bus are represented by $P_{load}^{j}$ and $Q_{load}^{j}$, respectively. The active and reactive powers delivered by *z*^th^ DG are represented by $P_{DG}^{Z}$ and $Q_{DG}^{Z}$ and the voltage at *j*^*th*^ bus are represented by *V*_*J*_.

## Proposed methodological framework

This section proposes a methodology for efficiently deploying the DG units in the grid-connected distribution networks using an AOA and SSA based hybrid optimization algorithm (AOASSA). Initially, the standard AOA and SSA are discussed individually. Then a hybrid model (AOASSA) has been presented in detail.

### Salp Swarm Algorithm (SSA)

The standard SSA is a population-based algorithm and was proposed by Mirjalili et al. [62]. The behavior of SSA can be demonstrated using a salp-chain searching for optimal food sources (i.e., the swarm’s target is a food source in the search space F). The salps are classified as leaders or followers in SSA based on their place in the chain. The chain begins with a leader and is followed by the followers to guide their movements. It starts by initializing the salp population, which is represented by the swarm X of n salps. The fitness of each salp is calculated to identify which salp has the best fitness (i.e., leader). The updated moment of leader position is obtained by Eq (7)

$$x_{i}^{1}=\{\begin{array}{c} y_{i}+c_{1}((Ub_{i}-Lb_{i})\times c_{2}+Lb_{i})\;c_{3}\geq 0.5\\ y_{i}-c_{1}((Ub_{i}-Lb_{i})\times c_{2}+Lb_{i})\;c_{3}<0.5 \end{array}$$ (7)

Where $x_{i}^{1}$ denotes the first position of the salp in the *i*^*th*^ dimension and *y*_*i*_ denotes the position of the food in the *i*^th^ dimension. The lower and upper bounds of the *i*^th^ dimension are denoted by *Lb*_*i*_ and *Ub*_*i*_, respectively. The coefficients *c*_2_ and *c*_3_ are randomly generated integers between 0 and 1, while the coefficient *c*_1_ is computed by Eq (8)

$$c_{1}=2e^{-{\left(\frac{4l}{L}\right)}^{2}}$$ (8)

Where l represents the current number of iteration and L represents maximum number of iterations. The term c_1_ is significant in SSA because it balances the exploration and exploitation during the entire search process. The position of the follower’s salps are represented by Eq (9).


$$x_{i}^{j}=\frac{\left(x_{i}^{j}-x_{i}^{\left(j-1\right)}\right)}{2}$$ (9)

The salps update their position based on population, and the program runs until the iteration count reaches its maximum. The entire working procedure can be visualized from its basic paper [48].

### Arithmetic Optimization Algorithm (AOA)

The AOA is a recently introduced population-based algorithm. Its standard variant was proposed by Abualigah et al. in 2021 [63]. The use of arithmetic operators (addition, subtraction, multiplication, and division) in solving optimization problems is the primary source of inspiration for AOA. It starts with initializing a set of random integers. Before starting with the use of mathematical operators, a feasible search space is selected with the help of coefficient Math operator accelerated (MOA) which is calculated by Eq (10).


$$MOA(C\_iter)=Min+C\_iter\frac{Max‐Min}{M\_iter}$$ (10)

Where MOA (C_iter) denotes the function value at the *t*^*th*^ iteration, as determined by Eq (10). The current iteration, which is between 1 and the maximum number of iterations (M_iter), is denoted by C_iter. The minimum and maximum values of accelerated functions are represented by Min and Max, respectively. According to the Arithmetic operators, mathematical computations using the Division (D) or Multiplication (M) operators results in high distributed values that commit to the exploratory search mechanism. Therefore, the division operator initially comes into action at the start of the search space due to its high dispersion in the search space. The multiplication operator follows the search mechanism, which has the second-highest dispersion in the search space. The updating equations for the position during the exploration phase are presented in Eq (11).


$$X_{i,j}(C_{\mathrm{iter}}+1)=\{\begin{array}{c} Bestx_{j}÷((MOP+\in )\times (Ub_{j}-Lb_{j})\times \mu Lb_{j})\;r_{2}<0.5\\ Bestx_{j}\times ((MOP+\in )\times (Ub_{j}-Lb_{j})\times \mu Lb_{j})\;otherwise \end{array}$$ (11)

Where *X*_*i*,*j*_(C_iter_+1) represents the *i*^*th*^ solution in the next iteration, *X*_*i*,*j*_(C_iter_) represents the *j*^*th*^ position of the *i*^th^ solution in the current iteration, and *Bestx*_*j*_ represents the *j*^th^ place in the best-obtained solution. The upper bound value and lower bound value of the *j*^th^ position are represented by *Ub*_*j*_ and *Lb*_*j*_, respectively. A number that works in the nested loop and balances division and multiplication operators during the exploration process in the search process is represented by *r*_2_. ∈ is a small integer number, μ is a control parameter for adjusting the search process. It is set at five with respect to test results obtained in the study. *MOP* is an exponentially decreasing function and can be obtained with Eq (12)

$$MOP(C\_iter)=1-\frac{C\_iter^{\frac{1}{\alpha }}}{M\_iter^{\frac{1}{\alpha }}}$$ (12)

In the exploitation phase, the solutions are updated with subtraction and addition operators, respectively. This is due to the fact that the subtraction operator observes the comparatively lower dispersion. Finally, the least dispersion is observed with the addition operator in the search space. The position updating equations are described in Eq (13).


$$X_{i,j}(C_{\mathrm{iter}}+1)=\{\begin{array}{c} Bestx_{j}-((MOP+\in )\times (Ub_{j}-Lb_{j})\times \mu Lb_{j})\;r_{3}<0.5\\ Bestx_{j}+((MOP+\in )\times (Ub_{j}-Lb_{j})\times \mu Lb_{j})\;otherwise \end{array}$$ (13)

Where r_3_ is a randomly generated integer which balances between subtraction and addition operators during the search process. The program continues to run until maximum iterations are reached, the coefficients MOA and MOP update their values, and four basic arithmetic operators explore, and exploit based on the extent of respective dispersions.

### Parallel operated hybrid Arithmetic Optimization Algorithm Salp Swarm Algorithm (AOASSA)

As per the no-free lunch theorem, no algorithm is feasible for all optimization problems. Every algorithm has few strengths and weaknesses that introduce an opportunity to either modify the basic version of the algorithm or hybridize two algorithms. In this paper, the active power losses are minimized with the developed parallel hybrid AOASSA. The prominent features and major differences amid individual algorithms (AOA and SSA) are discussed in Table 2.

**Table 2:** Prominent differences in features amid AOA and SSA.

Prominent Features	Salp Swarm Algorithm (SSA)	Arithmetic Optimization Algorithm (AOA)
Operational phenomenon	The population bifurcated into leaders and followers	The population is divided into division, multiplication, subtraction, and addition operators
Exploration and exploitation capabilities	The coefficient *c*_*1*_ is an exponentially decreasing function providing better exploration than MOP	Math Optimizer Probability (MOP) is also an exponentially decreasing function providing better exploitation than *c*_*1*_
Propulsion equation of particles	Balance of exploration and exploitation depends on *c*_1_	Balance of exploration and exploitation depends on MOA
Working principle	After initializing and sorting the best salps in the first iteration, the main loop runs. The Salp positions are updated based on the leader and follower equation; hence the program runs up to the maximum number of iterations.	After the initialization of solutions, MOA and MOP are updated. The main loop runs based on the value of MOA, enabling division and multiplication, subtraction and addition, solutions are updated up to the maximum number of iterations
Level of complexity	The convergence is based on leader, follower, and *c*_*1*_ equations making the algorithm architecture relatively simple.	The convergence is based on division, multiplication, subtraction, addition, MOA, and MOP equations making relatively a complex phenomenon
Strengths and weaknesses	The iterative parameter *c*_1_ provides better exploration but lacks exploitation. The sorting mechanism supports exploration search.	The iterative parameter MOP provides good exploitation but lacks exploration. The subtraction and addition operators provide relatively better exploitation

It can be concluded from Table 2 that both the algorithms (AOA and SSA) are capable enough to converge towards the optimal solution. The coefficient *c*_*1*_ in updating leader Salp position is an exponentially decreasing function with highly diversified values in exploration and less diversified in the exploitation phase, while MOP in AOA is also an exponentially decreasing function with comparatively lower divergence in the exploration phase and relatively lower divergence of particles in the exploitation phase, making SSA superior over AOA. As per the no-free lunch theorem, no algorithm is perfect in all problems, so a parallel operated hybrid combination of AOA and SSA is proposed to compare the best solutions in every run and store the best value after comparing it with upcoming run. The phenomenon will undoubtedly reduce the deficiencies of both algorithms.

**Fig 1:**
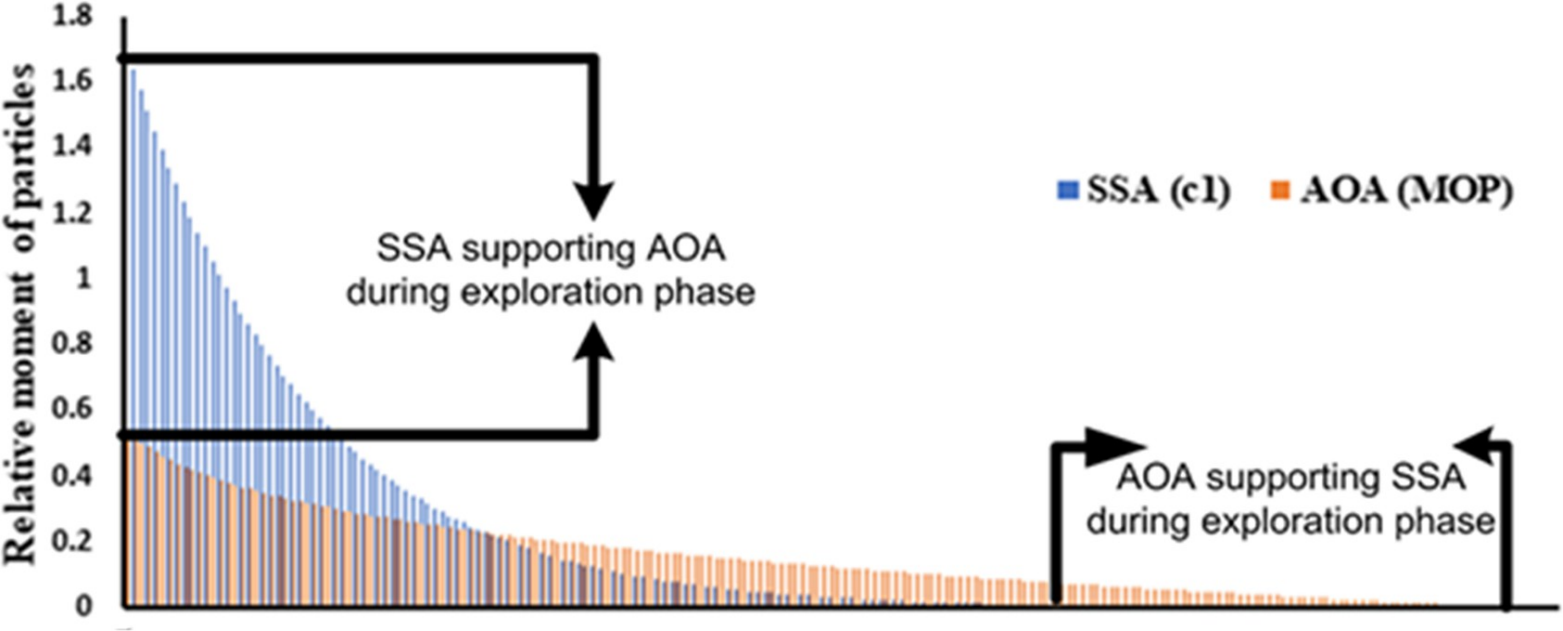
Comparison of position updating coefficients in SSA and AOA.

It is visualized from Fig 1 that the coefficients *c*_*1*_ and MOP are equally important during the search process. The coefficient c_1_ in SSA has good exploration capabilities at the beginning of the search process. The searching capability of *c*_*1*_ is fast as compared to MOP. In contrast, while the coefficient MOP has good exploitation capabilities at the end of the search process (however, the search process is comparatively slow). Thus, the hybrid AOASSA provides an opportunity to enhance the search capabilities during the entire search process.

**Fig 2:**
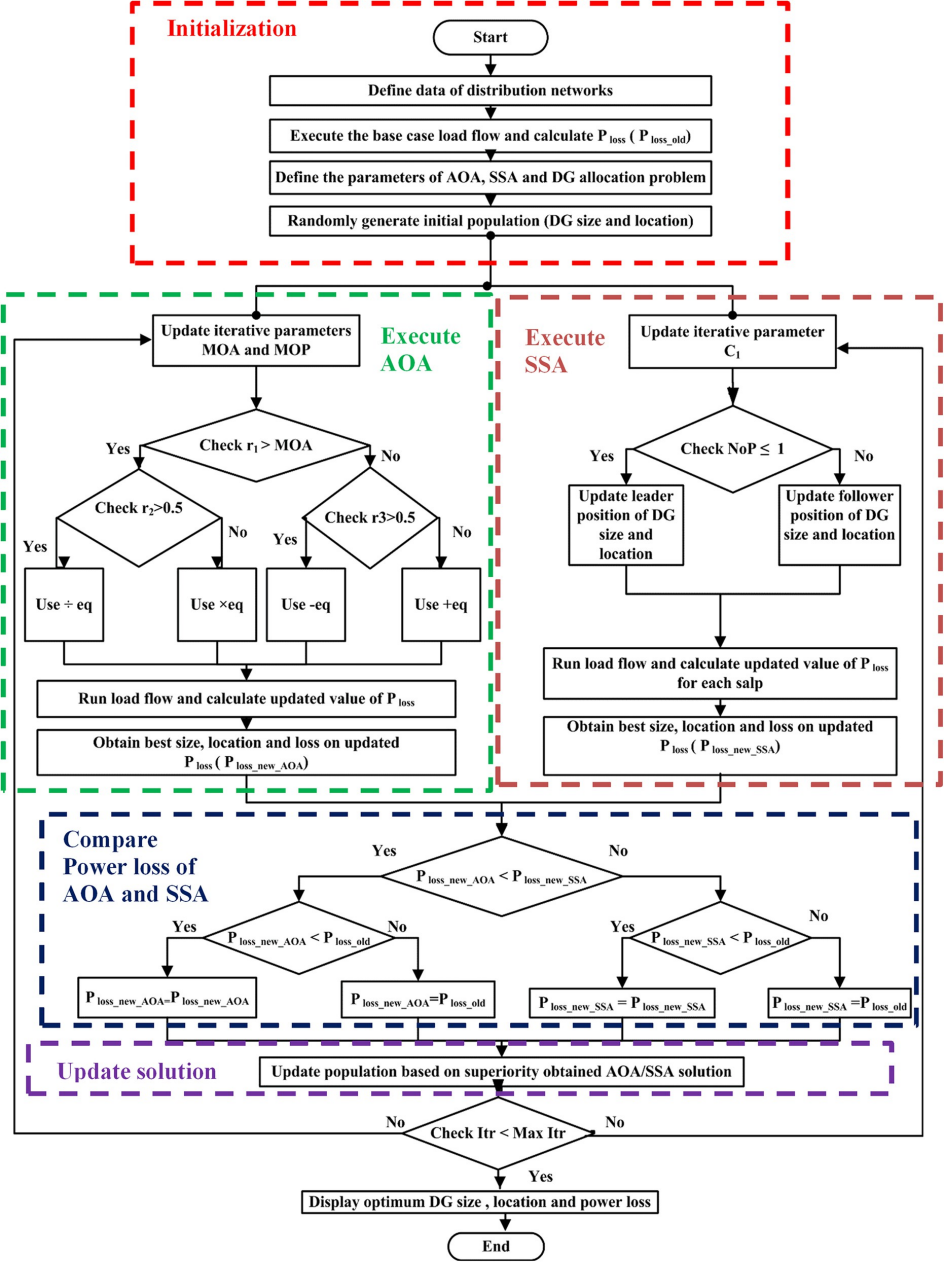
Flow chart of parallel operated hybrid AOASSA.

The flowchart of the proposed hybrid AOASSA for the optimal DG allocation problem is presented in Fig 2 and the steps are discussed below:

**Step 1:** Define the load data (branch resistance, reactance, conductance, and susceptibility. Active and reactive load on each node) as well as the line data (branch resistance, reactance, conductance, and susceptibility) for the 33-bus and 69-bus RDNs
**Step 2:** Run the base case load flow and calculate the initial power loss (termed as P_loss_old_) for the base case (without allocation of DG units)
**Step 3:** Define the parameters of AOA and SSA for the DG allocation problem. The parameters set for the optimization technique and DG allocation problem are presented in Table 3 [48], [49].

**Table 3:** Parameters of the optimization problem and hybrid AOASSA.

Parameters	Values
Population size (NoP)	50
Maximum number of iterations (Max_iter)	200
Lower bound for generator size (*Lb*_g_)	0
Upper bound for generator size (*Ub*_g_)	Total active load on the network
Lower bound for generator location (*Lb*_gc_)	2 (since bus 1 is a slack bus)
Upper bound for generator location (*Ub*_gc_)	33- or 69- (depending on network configuration)
Math operator probability maximum value (MOP_Max)	1
Math operator probability minimum value (MOP_Min)	0.2
Sensitive parameter (α)	5
Control parameter (μ)	0.5 (approx.)
c_2_, c_3_, *r*_1_ *r*_2_, *r*_3_	Random integers (0,1)

**Step 4:** Randomly initialize DG size and location within the search space as decision variables. The total number of decision variables is two (DG size and location). DG allocation problem with *x* potential solutions and *y* decision variables the element of population set *Z* will be presented as Eq (14)

$$Z=[\left|\begin{array}{ccc} P_{DG1,1} & P_{DG1,2\ldots \ldots \ldots ..} & P_{DG1,y}\\ P_{DG2,1} & P_{DG2,2\ldots \ldots \ldots \ldots } & P_{DG2,y}\\ \vdots & \vdots & \vdots \\ \vdots & \vdots & \vdots \\ \vdots & \vdots & \vdots \\ P_{DGx,1} & P_{DGx,2} & P_{DGx,y} \end{array}||\begin{array}{ccc} N_{DG1,1} & N_{DG1,2\ldots \ldots \ldots \ldots .} & N_{DG1,y}\\ N_{DG2,1} & N_{DG2,2\ldots \ldots \ldots \ldots .} & N_{DG2,y}\\ \vdots & \vdots & \vdots \\ \vdots & \vdots & \vdots \\ \vdots & \vdots & \vdots \\ N_{DGx,1} & N_{DGx,2\ldots \ldots .\ldots ..} & N_{DGx,y} \end{array}\right|]$$ (14)

**Step 5:** Execute load flow with AOA, update MOA and MOP with Eqs (10) and (12), respectively.
**Step 6:** Check, if the randomly generated integer *r*_1_ > MOA, then move towards the nested decision one and compare with *r*_2_. If *r*_2_ > 0.5, use division Eq (11) to update DG size and location, else use multiplication Eq (11) to update DG size and location for exploration. It must be noted that the coefficient MOA linearly increases from 0.2 to 0.9, and after few iterations, MOA exceeds *r*_1_ and MOA > *r*_1_, then move to decision two and compare *r*_3_. If *r*_3_ > 0.5, use subtraction Eq (13) to update DG size and location, else use addition Eq (13) to update DG size and location.
**Step 7:** After updating particles using Eqs (11) and (13), calculate the updated value of power loss (P_loss_)
**Step 8:** Obtain the best size/sizes, location/locations of DG, and the updated value of power loss obtained from AOA (termed as P_loss_new_AOA_)
**Step 9:** Execute the load flow with SSA, update the value of the iterative parameter *c*_1_ using Eq (8).
**Step 10:** Compare the population count; if the number of particles (NoP) is 1, use Eq (7) to update leader position of DG size and location; else update DG size and location with follower equation (refer to Eq 9).
**Step 11:** Run the load flow and calculate the power loss for each salp
**Step 12:** Sort out the salp population and obtain the best DG size and location that resulted in the least losses (termed as P_loss_new_SSA_)
**Step 13:** Compare step 8 and step 12, check if P_loss_new_AOA_ < P_loss_new_SSA_ than move to next check, and compare if P_loss_new_AOA_ < P_loss_old_, if again yes replace P_loss_new_AOA_ with P_loss_new_AOA_, if no, replace P_loss_new_AOA_ with P_loss_old_. On contrary, if the P_loss_new_SSA_ < P_loss_new_AOA_ move to next check, if P_loss_new_SSA_ < P_loss_old_, replace P_loss_new_SSA_ with P_loss_new_SSA_, else replace P_loss_new_SSA_ with P_loss_old_.
**Step 14:** Update the population based on the superiority of the solution. Replace the DG sizes and locations with the solutions obtained from the least P_loss_.
**Step 15:** Repeat the program until it reaches the predefined maximum iterations count.

**Fig 3:**
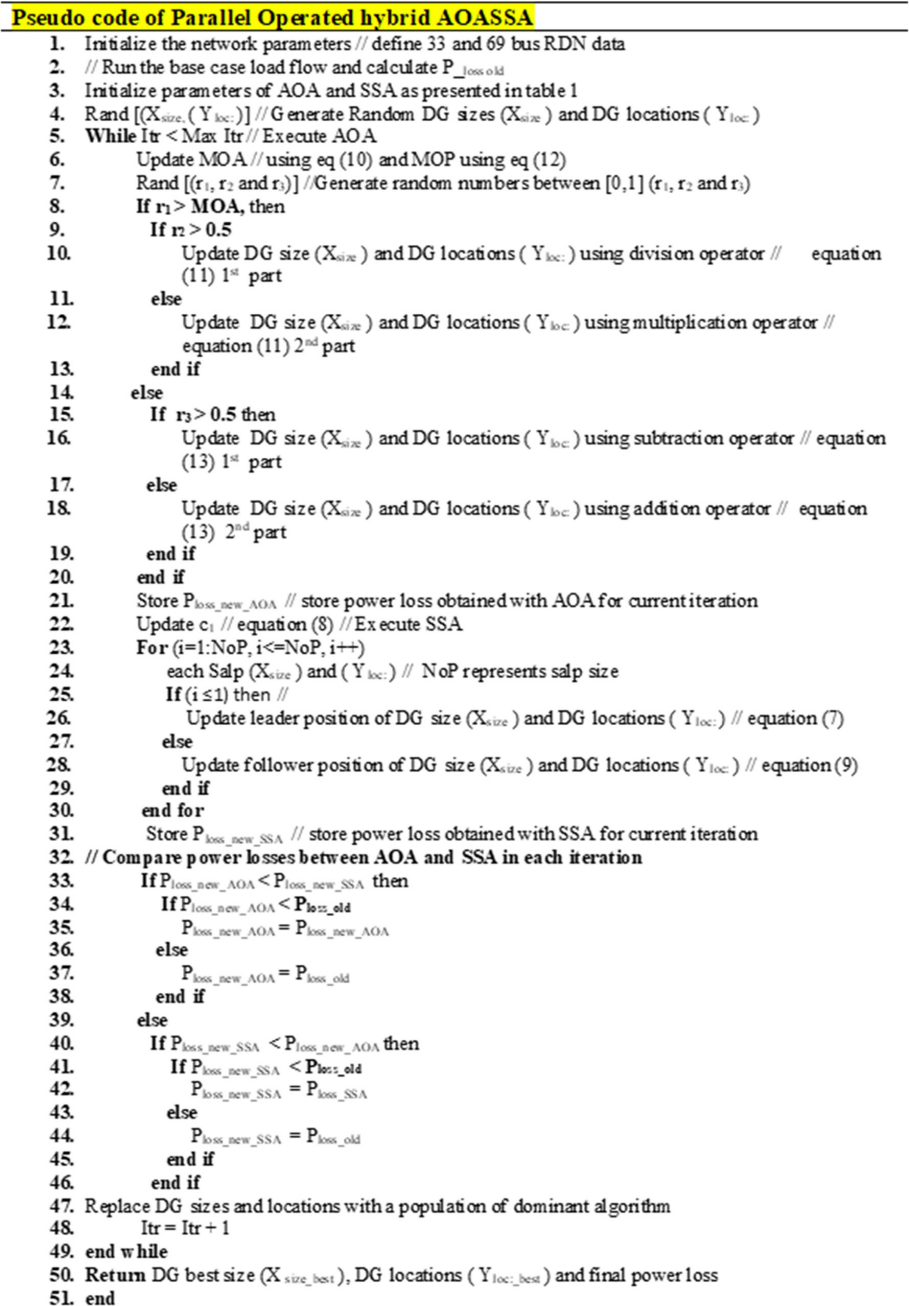
Pseudo code of parallel operated hybrid AOASSA.

The detailed Pseudo code of proposed parallel operated hybrid AOASSA is presented in Fig 3

## Results and discussions

The standard radial test systems (IEEE 33-node and 69-node) are used to implement the proposed AOASSA algorithm. In addition to the AOASSA, the hybrid SSAPSO, AOAPSO, and respective standard algorithms (AOA, SSA, and PSO) are implemented to compare different results. The findings of many recent optimization approaches were obtained from literature for comparison and validation. The basic case refers to the examination of networks that do not have any compensating devices (without allocation of DG units). The AOASSA is implemented in the MATLAB 2017 environment. The study has been classified on three cases based on the number of DG units allocated, as shown in Table 4. The analysis on varying penetration of DGs revealed the performance analysis of the proposed algorithm (AOASSA) at different dimensions (number of DG units allocated). All simulations run on a laptop with an Intel Core i5 4th Generation Intel(R) Core(TM) i5-4210U CPU @ 1.70GHz 2.40 GHz, an SSD drive, and a 64-bit operating system.

**Table 4:** Cases based on number of DG allocation units.

Cases	Case description
Base case	With no DG allocation
Case 1	Optimal siting and sizing of a single DG unit
Case 2	Optimal siting and sizing of two DG units
Case 3	Optimal siting and sizing of three DG units

### 33-Bus Radial Distribution Network (RDN)

**Fig 4:**
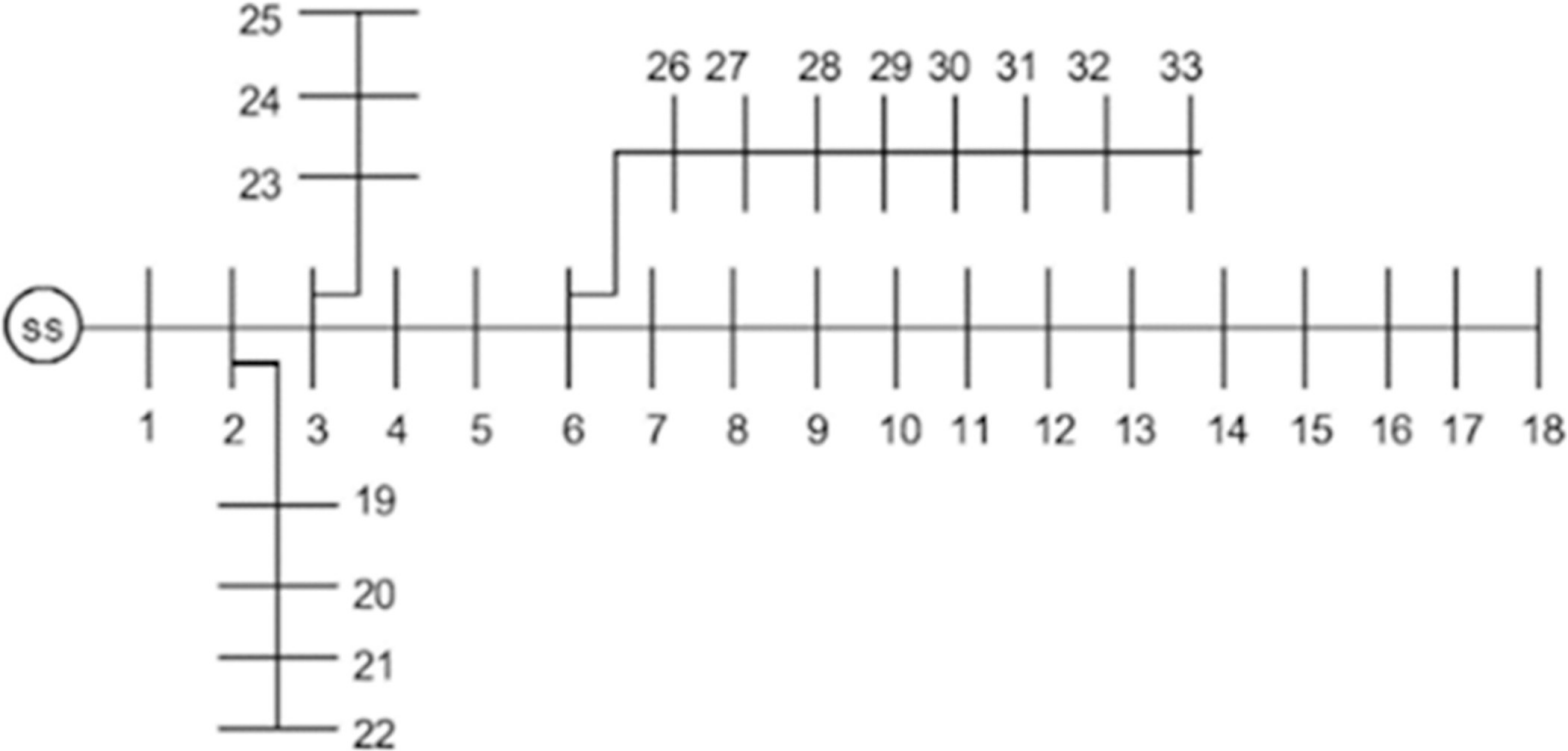
IEEE 33-Bus radial distribution network.

The 33-bus RDN has an active and reactive load of 3.715 MW and 2.3 MVArs, respectively. The network receives 12.66 kV from a step-down transformer linked to node 1. The losses for 33-bus RDNs without allocation of DG units are 211 kW and 143.12 kVAR, respectively, with a minimum voltage of 0.90 p.u recorded at node 18 in the base scenario. The network structure of 33-bus RDN is presented in Fig 4.

### Power loss minimization in 33-bus RDN

The decision or design variables are the quantities that decision-makers can control or change. These parameters must be optimized to obtain the required objective function values. In the proposed grid-connected DG allocation study, the design or decision variables are DG size and DG location, and the objective function is to minimize the real power losses (P_loss_). Initially, the standard AOA, SSA and PSO are applied to optimize sizes and locations that result in the least losses. The AOA performs better than SSA and PSO, as evident from Table 4. At the same time, SSAPSO, AOAPSO, and AOASSA exhibit the same characteristics under low dimensional problems (with the allocation of 1 DG unit). Furthermore, the AOA outcomes indicate that a DG sized 2373.6 kW at bus 26, SSA outcomes indicate that 2186.1 kW DG installed at bus 26, and PSO outcomes indicate that a DG sized 2115.4 kW placed at bus 26 results in minimized losses. On the other hand, the hybrid SSAPSO, AOAPSO, and AOASSA show that optimal size and location in the 33-bus RDN is 2592.5 kW at bus 6, respectively. In case 2, the AOA outcomes indicate that 1031.5kW DG at bus 30 and 865.72kW at bus 13, SSA outcomes indicate that 1180.2 kW at bus 30 and 780.33 kW DG at bus 15, and PSO outcomes indicate that a DG of 893.63 kW and 1042.6 kW installed at bus 31 and 12, respectively result in most minimized losses. On the contrary, the hybrid SSAPSO show that the optimal size and location of two DG units in 33 bus radial distribution network is 851.94 kW at bus 13 and 1158.5 kW at bus 30, respectively. The AOAPSO and AOASSA suggest the same DG size and location in case 2 (i.e., 1158.50 kW and 851.94 kW to be placed at buses 30 and 13, respectively). In case 3, complexity is increased compared to cases 1 and 2 due to the simultaneous allocation of three DG units. It is evident from Table 5 that the AOA produces DG sizes and locations that results in the least losses compared to SSA and PSO individually; however, the power losses are still greater than the three hybrid combinations. The hybrid AOASSA suggested DG sizes and locations that result in better performance than SSAPSO. However, the performance of the proposed AOASSA is the same as AOAPSO due to the higher diversification produced by PSO’s linearly decreasing inertia weight at the start of the search phase. In contrast, MOP, subtraction, and addition operators produce lesser diversification in exploitation phase. Therefore, the AOA outperforms SSA and PSO in all three cases by producing more PLR. The diversity of particles produced by multiplication and division operators in the exploration phase and subtraction/addition operators in the exploitation phase helped to avoid local optima stagnation. However, the exploration capabilities of AOA are still lacking due to relatively less diversification produced by MOP at the start of the search process. The exploration capabilities are later improved with SSA (*c*_1_ operator), which avoided local optima stagnation and produced DG sizes and locations, resulting in the least losses. The performance of the proposed AOASSA with the allocation of multiple DG units is shown in Table 5.

**Table 5:** Performance of proposed AOASSA on 33 Bus RDN with multiple DGs.

Case	Optimization Technique	DG Size, kW (@Bus location)	Power loss (kW)	PLR (%)
Base Case	-	-	211.00	-
Case 1	AOA	2373.6(26) 2186.1(26) 2115.4(26) 2592.5(6) 2592.5(6) 2592.5(6)	113.01	46.44
	SSA		113.98	45.98
	PSO		114.63	45.67
	SSAPSO		111.01	47.39
	AOAPSO		111.01	47.39
	AOASSA		111.01	47.39
Case 2	AOA	1031.5(30), 865.72(13) 1180.2(30), 780.33(15) 893.63(31), 1042.6(12) 851.94(13), 1158.5(30) 1158.5(30), 851.93(13) 1158.5(30), 851.93(13)	87.65	58.46
	SSA		88.17	58.22
	PSO		88.98	57.83
	SSAPSO		87.16	58.69
	AOAPSO		87.16	58.69
	AOASSA		87.16	58.69
Case 3	AOA	898.82(24), 807.77(13), 1078.2(30) 798.51(25), 797.25(13), 1020(30) 829.02(25), 724.08(15), 928.89(31) 879(13), 1076.3(24), 1018.8(30) 1054.3(30), 1093.9(24), 802.01(13) 1054.3(30), 802.01(13), 1093.9(24)	73.22	65.30
	SSA		73.76	65.04
	PSO		76.02	63.97
	SSAPSO		73.07	65.37
	AOAPSO		72.78	65.51
	AOASSA		72.78	65.51

### Convergence quality analysis

**Fig 5:**
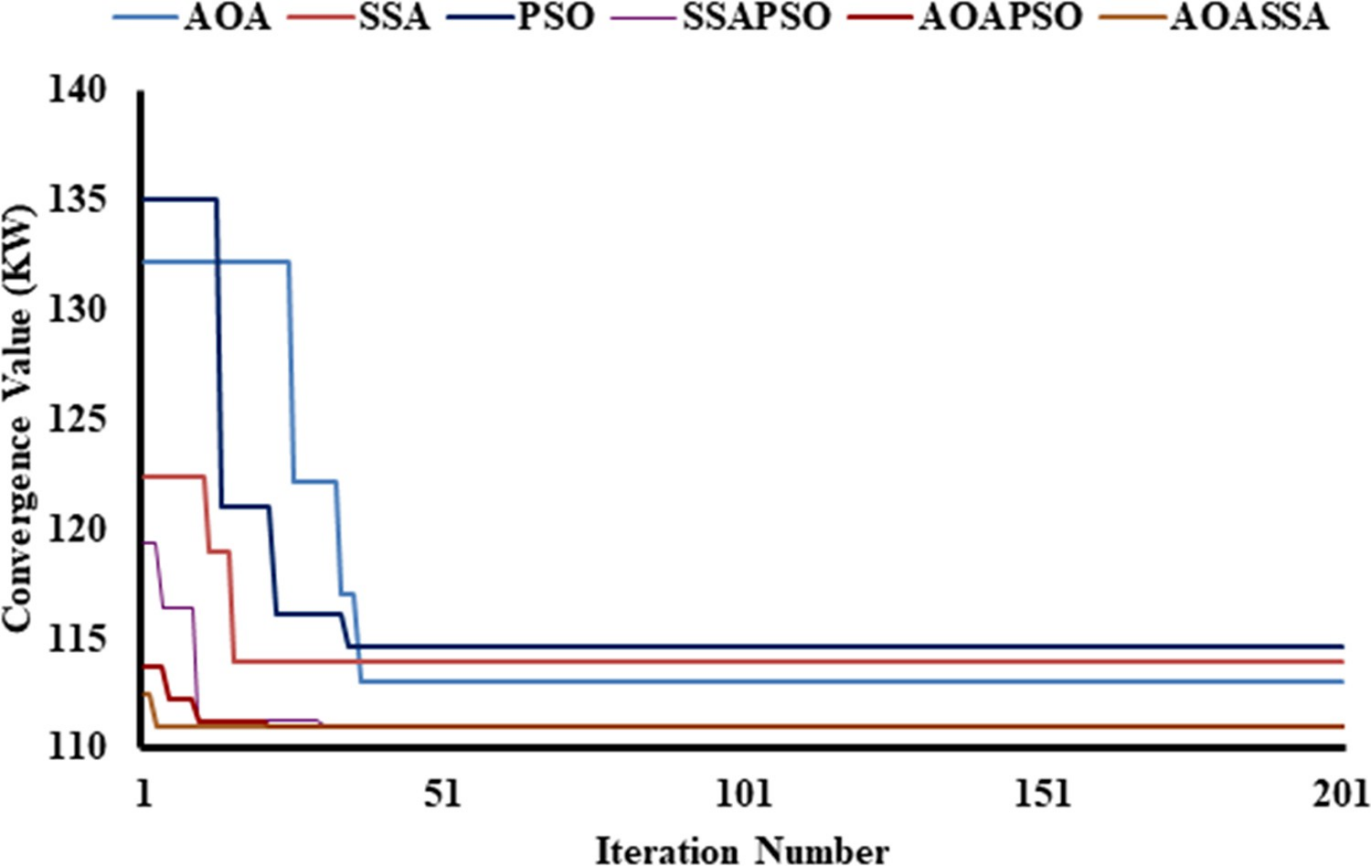
Convergence characteristics of contending optimization techniques for optimal allocation of single DG unit in the 33-bus RDN.

The proposed AOASSA produces a superior quality solution dealing with high-dimensional DG allocation problems. A high solution diversity at the beginning of the search mechanism (exploration phase) and lower diversity at the end of the search mechanism (exploitation phase) is required to produce quality solutions. It is evident from Fig 5 that the SSA has better exploration capabilities than AOA and PSO when algorithms are compared individually. In contrast the exploitation capabilities of AOA are superior to contending algorithms in case 1. The AOA reduced the power losses from 211 kW to 132.1 kW (37.39%), SSA reduced power loss to 122.4 kW (41.99%) in the first iteration. In comparison, PSO reduced the power losses to 135 kW(36.01%) in the first iteration, addressing the superiority of SSA in the exploration phase (due to higher diversification produced by *c*_*1*_) at the start of the search phase. On the other hand, AOA converged to 113.01 kW (46.44%), SSA converged to 113.98 kW (45.98%), and PSO converged to 114.63 kW (45.67%), highlighting the exploitation capabilities of AOA due to denser solutions produced by AOA (subtraction, addition operator, and denser exponential decrease of MOP). AOASSA has good exploration and exploitation characteristics when dealing with a single DG unit’s allocation unit (case 1). The allocation of one or two DG units is relatively less complex than the optimal allocation of three DG units as the complexity of the search space increases. The dominance of AOASSA against its counterparts (SSAPSO and AOAPSO) in terms of exploration and exploitation is evident in Fig 5.

The SSAPSO and AOAPSO have chosen a search space which results in poor exploration as compared to AOASSA. The SSAPSO reduces the losses from 211 kW to 119.33 KW (43.44%), the AOAPSO reduced the losses from 211 kW to 113.68 kW (46.12%). The proposed AOASSA reduced the losses from 211 kW to 112.45 kW (46.70%) in the first iteration while dealing with the allocation of one DG unit. Later in the exploitation phase, SSAPSO, AOAPSO, and AOASSA converged to the same power loss value but with different DG sizes at different locations. The iterative parameter in SSA supports the exploration capabilities, while the iterative parameter in AOA supports the exploitation capabilities in the search process. The results demonstrate that the exploration capabilities of AOASSA are superior to its counterpart while the exploitation capabilities are same compared to counterparts. Due to less complexity, while dealing with single DG unit allocation, hybrid SSAPSO, AOAPSO, and AOASSA converge to the same power loss value.

**Fig 6:**
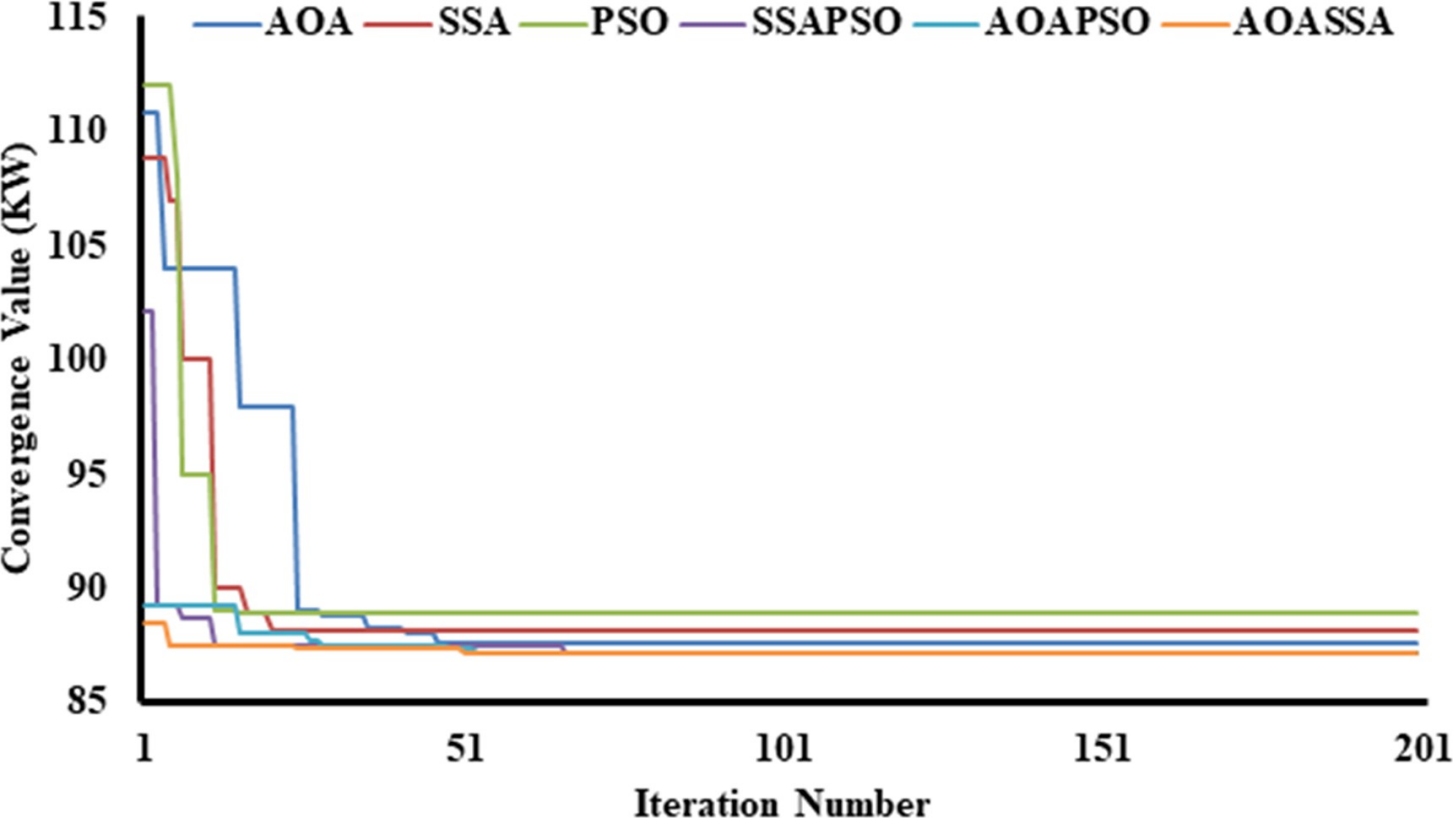
Convergence characteristics of contending optimization techniques for optimal allocation of two DG units in the 33-bus RDN.

Similarly, while dealing with the allocation of two DG units, the AOA reduced the power loss from 211 kW to 110.87 kW (47.45%), SSA reduced power loss to 107 kW (49.29%), and the PSO reduced the power loss to 112 kW (46.91%), addressing the superiority of SSA in terms of exploration capabilities, as shown in Fig 6. AOA leads in the exploitation phase, selecting the appropriate region with minimum diversification of particles. As a result, the AOA converged to 87.65 kW (58.46%) SSA converged to 88.17 kW (58.21%), and PSO converged to 88.98 kW (57.83), highlighting the superiority of AOA in the exploitation phase. In terms of hybrid algorithms, the proposed AOASSA performed better in exploration and exploitation capabilities. The SSAPSO reduces the losses from 211 kW to 102.14 kW (51.59%), the AOAPSO reduced the losses from 211 kW to 89.23 kW (57.71%), and the proposed AOASSA reduced the losses from 211 kW to 88.48 kW (58.07%) in the first iteration while dealing with the allocation of two DG units. Furthermore, the SSAPSO, AOAPSO, and AOASSA converged to the same power loss value (i.e., 87.16 kW equals 58.69%).

**Fig 7:**
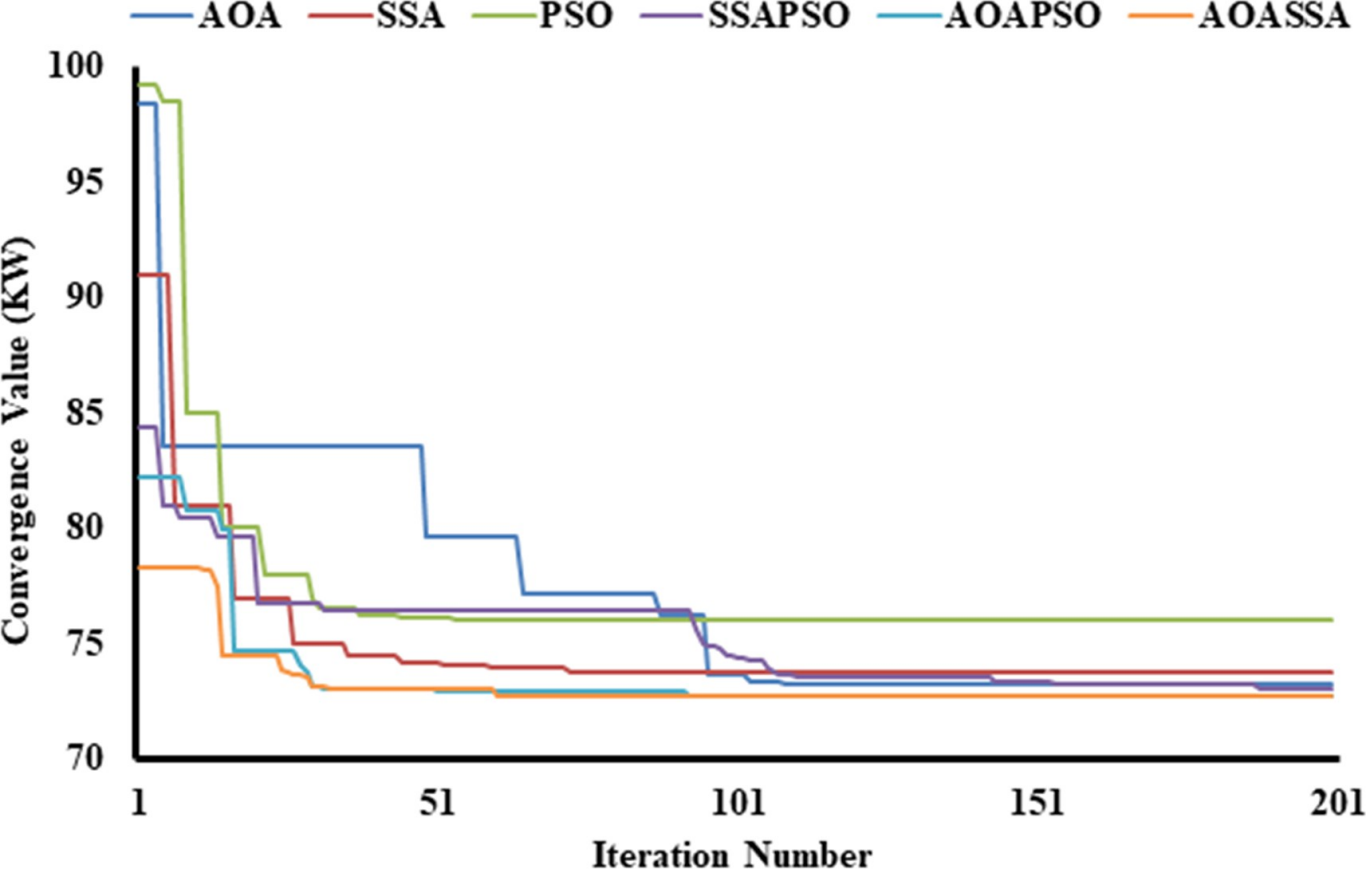
Convergence characteristics of contending optimization techniques for optimal allocation of three DG units in the 33-bus RDN.

In the third case, while dealing with the allocation of 3 DG units, the AOA converged to 98.41 kW (53.36%), SSA converged to 91 kW(56.87%), and PSO converged to 99.2 kW (52.99%), highlighting the exploration capabilities of SSA at the beginning of the search process. At the end of the search process, the AOA leads SSA and PSO in solution convergence. The AOA reduced the power loss to 73.22kW (65.30%), SSA reduced the power loss to 73.76kW (65.04%), and PSO reduced the power loss to 76.02kW (63.97%), highlighting the exploitation capabilities of AOA. In terms of hybrid algorithms, the proposed AOASSA performs better in exploration and exploitation capabilities. The SSAPSO reduces the losses from 211 kW to 84.38 kW (60.01%), the AOAPSO reduced the losses from 211 KW to 82.19 kW (61.05), and the proposed AOASSA reduced the losses from 211 kW to 78.3 kW (62.89%) in the first iteration while dealing with the allocation of three DG units. It must be noted that the proposed AOASSA has superior exploitation capabilities compared to SSAPSO, which stuck to local optima when three DG units were allocated, resulting in higher losses compared to its counterparts. However, the exploitation capabilities of AOASSA are similar to AOAPSO as the power losses obtained by AOAPSO and AOASSA are the same at the end (i.e., 72.78 kW equals to 65.51%). The proposed AOASSA aggregately takes the benefit of the iterative parameter of SSA (*c*_*1*_) during the exploration phase, producing high divergence at the beginning of the search process. On the other hand, MOP, subtraction, and addition operator at the end of the search process enhances the exploitation capabilities of the AOASSA. The unique search mechanism of AOASSA can be observed in the case of three DG units allocations, which leads to avoiding the loss of diversity, resulting in more accurate and précised solutions. The convergence superiority of the proposed AOASSA has been presented in Fig 7.

### Convergence speed analysis

The convergence speed is purely dependent on the diversity of the particles in the search space and is measured in terms of number iterations count to converge towards the best solution. The lesser the number of iterations to reach the best solutions, the faster the algorithm. The convergence speed also depends on the complexity of the problem. In terms of DG allocation, increasing the number of DG unit’s allocation increases the complexity of the problem (due to increment in search space). As discussed, the AOASSA support each other during exploration and exploitation; the solutions quickly converge towards the optimal solution. In case1, AOA is comparatively slower than SSA and PSO; AOA converges the best solution in 37 iterations, SSA converges in 16 iterations, and PSO converges in 14 iterations while dealing with the optimal allocation of one DG unit. In terms of hybrid algorithms, the AOASSA achieves the best DG size and location to obtain the least power losses in 3 iterations, followed by AOAPSO, which achieves optimal DG size and location to obtain the least power losses in 21 iterations. In comparison, SSAPSO takes 30 iterations to attain the best DG size and location, resulting in the least losses. In case2, AOA converges in 47 iterations, SSA converges in 21 iterations, and PSO converges in 17 iterations. For hybrid algorithms, 51 iterations are required to obtain the optimal DG sizes and locations by AOASSA, followed by AOAPSO that takes 53 iterations. The SSAPSO converged to the best solution in 67 iterations. Furthermore, in case3, AOA converged to the optimal solution in 109 iterations, SSA converged in 73 iterations, and PSO converged in 54 iterations. Among hybrid algorithms, AOASSA requires 61 iterations to obtain optimal DG size and location that produce minimum loss value, followed by AOAPSO that takes 93 iterations. The SSAPSO converged in 181 iterations to obtain a minimum losses value. As discussed, the AOASSA and AOAPSO converge to the same power loss value; however, the AOASSA is faster in response as compared to AOAPSO due to the excellent diversity of particles. It must be noted that the number of iterations to reach the best solution increase as the DG allocation units increase. It can be observed from Table 6 that AOA is comparatively slower than SSA and PSO (in all three cases) due to loss of diversity (premature convergence in the case of SSA and PSO). It must be noted that the SSA and PSO are un-doubtfully fast but have the problem of local optima stagnation and produce less quality solutions compared to AOA. The AOASSA outperforms in terms of convergence speed and achieve global best solution due to the superior exploration and exploitation capabilities.

**Table 6:** Convergence speed of proposed algorithms with multiple DG allocation units.

Case	Technique	Minimum iteration to reach global best
Case 1	AOA	37
	SSA	16
	PSO	14
	SSAPSO	30
	AOAPSO	21
	AOASSA	3
Case 2	AOA	47
	SSA	21
	PSO	17
	SSAPSO	67
	AOAPSO	53
	AOASSA	51
Case 3	AOA	109
	SSA	73
	PSO	54
	SSAPSO	181
	AOAPSO	93
	AOASSA	61

### Statistical analysis

The MHTs are stochastic in nature, and the reliability of an algorithm or performance superiority has been evaluated based on its mean, variance, and standard deviation value. For the DG allocation problem, six algorithms (AOA, SSA, PSO, SSAPSO, AOAPSO, and AOASSA) have been executed 30 times individually to test the reliability of the solutions obtained. The AOA offers the least mean, variance and standard deviations when MHTs are compared individually due to the search mechanism (AOA providing comparatively higher divergence at the start of the search process and denser solutions in the end); however, the inherent exploration capabilities lack due to the iterative parameter (MOP). Therefore, a hybrid combination of AOA with SSA diminishes the inherent exploration deficiencies of AOA. The proposed AOASSA has a lesser mean, variance, and standard deviation (based on best and worst values obtained in each case during 30 runs), as evident from Table 7. The AOASSA has superior statistical characteristics because the proposed AOASSA has a better exploration and exploitation mechanism, making it comparatively less dependent on the initial population. The exceptional exploration mechanism provided by SSA (iterative parameter c_1_) further followed by exploitation capabilities of AOA(iterative parameter MOP) makes the proposed AOASSA comparatively less dependent on initial population generated and guarantees low mean, variance, and standard deviations. Therefore, the proposed AOASSA is found to exhibit promising statistical characteristics as compared to its counterpart. The mean, variance, and standard deviations for the proposed AOASSA and its counterparts (AOAPSO and SSAPSO and respective individual algorithms) are presented in Table 7.

**Table 7:** Statistical superiority of proposed AOASSA.

Case Number	Technique	Mean Power Loss (kW)	Standard Deviation	Variance
Case 1	AOA	115.75	3.42	11.73
	SSA	118.64	4.17	17.36
	PSO	118.82	5.03	25.30
	SSAPSO	112.95	1.44	2.06
	AOAPSO	112.76	1.18	1.39
	AOASSA	111.29	0.73	0.53
Case 2	AOA	91.94	3.75	14.03
	SSA	92.00	4.68	21.95
	PSO	94.51	5.07	25.65
	SSAPSO	89.68	1.66	2.76
	AOAPSO	88.36	1.38	1.90
	AOASSA	87.74	0.98	0.96
Case 3	AOA	78.59	3.98	15.81
	SSA	82.14	4.77	22.77
	PSO	83.40	6.00	36.02
	SSAPSO	74.79	1.92	3.70
	AOAPSO	74.68	1.84	3.39
	AOASSA	73.46	1.19	1.41

### Cost analysis

**Fig 8:**
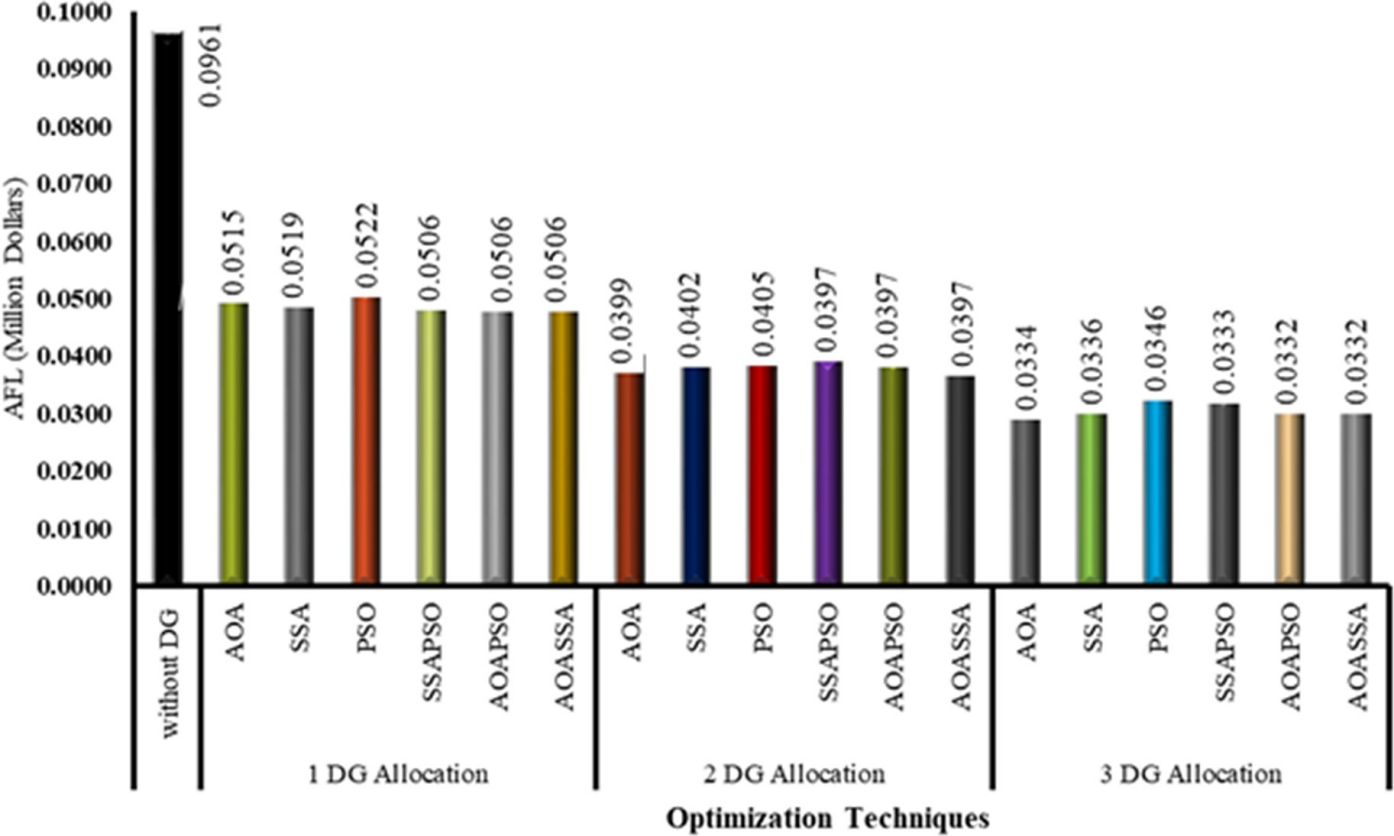
Annual financial losses for three cases with different optimization techniques.

The primary goal of developing the proposed AOASSA is to choose the optimal bus placement and size of DG to minimize power losses. The decrease in losses also lowers operational costs and aids grid reinforcement. In addition, the reduction in losses also translates to monetary savings in terms of cost. For this study, the per-unit cost of electricity (price/kWh) is assumed as 0.2121MYR, which is equal to $0.052. The initial cost of losses without DG allocation has been found to be 0.0961 million dollars. The optimal sizes and locations produced by AOA, SSA, and PSO reduces the power losses to 46.44%, 45.98%, and 45.67%, which reduces the AFL from 0.09611 million dollars to 0.0515, 0.0519, and 0.0522 million dollars, respectively. The optimal allocation of a single DG unit reduced the losses up to 47.39% (for SSAPSO, AOAPSO, and AOASSA), which reduced the proportional cost of losses. The cost of losses has been reduced from 0.0961 million dollars to 0.0506 million dollars with the optimal allocation of one DG unit irrespective of the optimization technique (in the case of hybrid SSAPSO, AOAPSO, and AOASSA). In case 2, the optimal sizes and locations of 2 DG units produced by AOA, SSA, and PSO reduces the power losses to 58.46%, 58.22%, and 57.83%, which in turn reduced the AFL from 0.0961 million dollars to 0.0399, 0.0402, and 0.0405 million dollars, respectively. Furthermore, using hybrid techniques (SSAPSO,AOAPSO and AOASSA), the optimal allocation of 2 DG units reduced the losses to 58.69%, reducing the financial losses with the same ratio. The cost of losses reduced from 0.0961million dollars to 0.0397 million dollars irrespective of the optimization technique applied. In case 3, the optimal sizes and locations of three DG units produced by AOA, SSA, and PSO reduces the power losses to 65.30%, 65.04%, and 63.97%, which declined the AFL from 0.09611 million dollars to 0.0334, 0.0336, and 0.0346 million dollars, respectively. However, with the optimal allocation of 3 DG units, the power losses were reduced to 65.37% with SSAPSO. In contrast, the power losses have been reduced to 65.51% for AOAPSO and AOASSA as compared to the base case. Therefore, the proportional cost of losses has been reduced from 0.0961 million dollars to 0.0333 million dollars for SSAPSO. The cost of losses reduced from 0.0961 million dollars to 0.332 million dollars for AOAPSO and AOSSA. The cost of losses for all three cases is depicted in Fig 8.

### Benchmarking power losses on 33-bus distribution network

**Fig 9:**
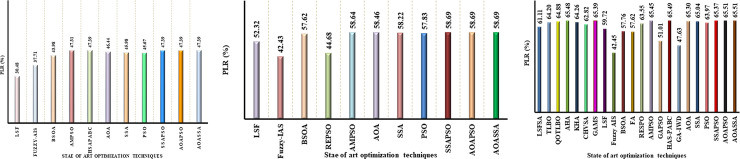
Comparative analysis of the AOASSA against the competitive optimization algorithms for the optimal allocation of (a) Single DG unit, (b) two DG units, (c) three DG units in the 33-bus RDN.

In order to validate the superiority of the proposed AOASSA, benchmarking against recent state of the art meta-heuristic algorithms has been presented. The optimal size, locations and PLR obtained by some existing state of the art MHTs presented in Table 8. A summary showing power loss reductions with multiple DG units obtained from literature has been illustrated in Fig 9A, 9B and 9C. The proposed AOASSA has superior results in terms of PLR, as shown in Fig 9A, 9B and 9C.

**Table 8:** Optimal capacities and positions of DGs in the 33-bus distribution network for proposed and benchmarked algorithms.

Optimization Techniques	1 DG		2 DGs		3 DGs	
	DG Size (kW), @Bus	PLR (%)	DG Size (kW), @Bus	PLR (%)	DG Size (kW), @Bus	PLR (%)
LSFSA [51]	-	-	-	-	1112.4(6) 487.4(18) 867.9(30)	61.11
TLBO [34]	-	-	-	-	824.6(10) 1031.1(24) 886.2(31)	64.20
QOTLBO [34]	-	-	-	-	880.8(12), 1059.2(24) 1071.4(29)	64.88
Algorithmic Heuristic Approach (AHA) [64]	-	-	-	-	792(13) 1068(24) 1027(30)	65.48
KHA [35]	-	-	-	-	810.7(13) 836.8(25) 841(30)	64.26
General Algebraic Modeling system (GAMS) [7]	-	-	-	-	755(14) 1073(24) 1068(30)	65.39
Loss Sensitivity Factor (LSF) [29]	743(18)	30.48	720(18).900(33)	52.32	720(18), 810 (33), 900(25)	59.72
Fuzzy AIS [36]	1931(32)	37.71	383.6(32) 1150.6(30)	42.43	2071(32) 111.38(30) 150.3(31)	42.45
Back tracking Searching Optimization Algorithm (BSOA) [65]	1858(8)	43.98	880(13).924(31)	57.62	632(13) 486(28) 550(31)	57.76
Firefly Algorithm (FA) [65]	-	-	-	-	652(14) 198.4(18) 1067.2(32)	57.62
REPSO [53]	-	-	1483.0(30) 383.6 (32)	44.68	1227.4(6) 606.8(14) 687(31)	63.55
Analytical method particle swarm optimization (AMPSO) [48]	2490(6)	47.31	830(13) 1110 (30)	58.64	790(13) 1070(24) 1010(30)	65.45
GAPSO [52]	-	-	-	-	925(11) 863(16) 1200(32)	51.01
Hybrid Harmony Search Algorithm and Particle Artificial Bee Colony (HSAPABC) [55]	2598(6)	47.39	-	-	755(14) 1073(24) 1068(30)	65.49
GAIWD [54]	-	-	-	-	1221.4(11) 683.3(16) 1213.5(32)	47.63
AOA	2373.6(26)	46.44	1031.5(30) 865.72(13)	58.46	898.82(24) 807.77(13) 1078.2(30)	65.30
SSA	2186.1(26)	45.98	1180.2(30) 780.33(15)	58.22	798.51(25) 797.25(13) 1020(30)	65.04
PSO	2115.4(26)	45.67	893.63(31) 1042.6(12)	57.83	829.02(25) 724.08(15) 928.89(31)	63.97
SSAPSO	2592.5(6)	47.39	851.94(13) 1158.9(30)	58.69	879(13) 1076.3(24) 1018.8(30)	65.37
AOAPSO	2592.5(6)	47.39	1158.5(30) 851.93(13)	58.69	1054.3(30) 1093.9(24) 802.01(13)	65.51
**AOASSA (Proposed)**	**2592.5(6)**	**47.39**	**1158.5(30) 851.93(13)**	**58.69**	**1054.3(30) 802.01(13) 1093.9(24)**	65.51

The PLR obtained with some recent state-of-the-art algorithms are depicted in Fig 9A, 9B and 9C. Initially, the results are benchmarked under low dimensions (1 DG unit allocation). While allocating single DG unit, the PLR obtained by the LSF method [29] is 30.48%, Fuzzy AIS [36] is 37.71%, BSOA [65] is 43.98%, AMPSO [48] is 47.31%, HSA-PABC [55] is 47.39%, AOA is 46.44%, SSA is 45.98%, PSO is 45.67%, SSAPSO is 47.39%, AOAPSO is 47.39% and proposed AOASSA is 47.39%. The results are then benchmarked for two DG units allocation. While allocating two DG units, the PLR obtained by LSF [29] is 52.32%, Fuzzy AIS [36] is 42.43%, BSOA [65] is 57.62%, RESPO [53] is 44.68%, AMPSO [48] is 58.64%, AOA is 58.46%, SSA is 58.22, PSO is 57.83, SSAPSO is 58.69%, AOAPSO is 58.69% and AOASSA is 58. 69%. In the end, the results are benchmarked for the allocation of three DG units. The power loss reduction obtained by LSFSA [51] is 61.11%, TLBO [34] is 64.20%, QOTLBO [34] is 64.88%, AHA [64] is 65.48%, KHA [35] is 64.26%, GAMS [7] is 65.39%, LSF [29] is 59.72%, Fuzzy AIS [36] is 42.45%, BSOA [65] is 57.76%, FA [65] is 57.62%, RESPO [53] is 63.55%, AMPSO [48] is 65.45%, GAPSO [52] is 51.01%, HSAPABC [55] is 65.49%, GAIWD [54] is 47.62%, AOA is 65.30%, SSA is 65.04%, PSO is 63.97%, SSA-PSO is 65.37%, AOAPSO is 65.51%, and AOASSA is 65.51%. The AOASSA has superior performance under all three cases due to the parallel combination of AOASSA supporting each other during the exploration and exploitation phase.

### 69-Bus Radial Distribution Network (RDN)

**Fig 10:**
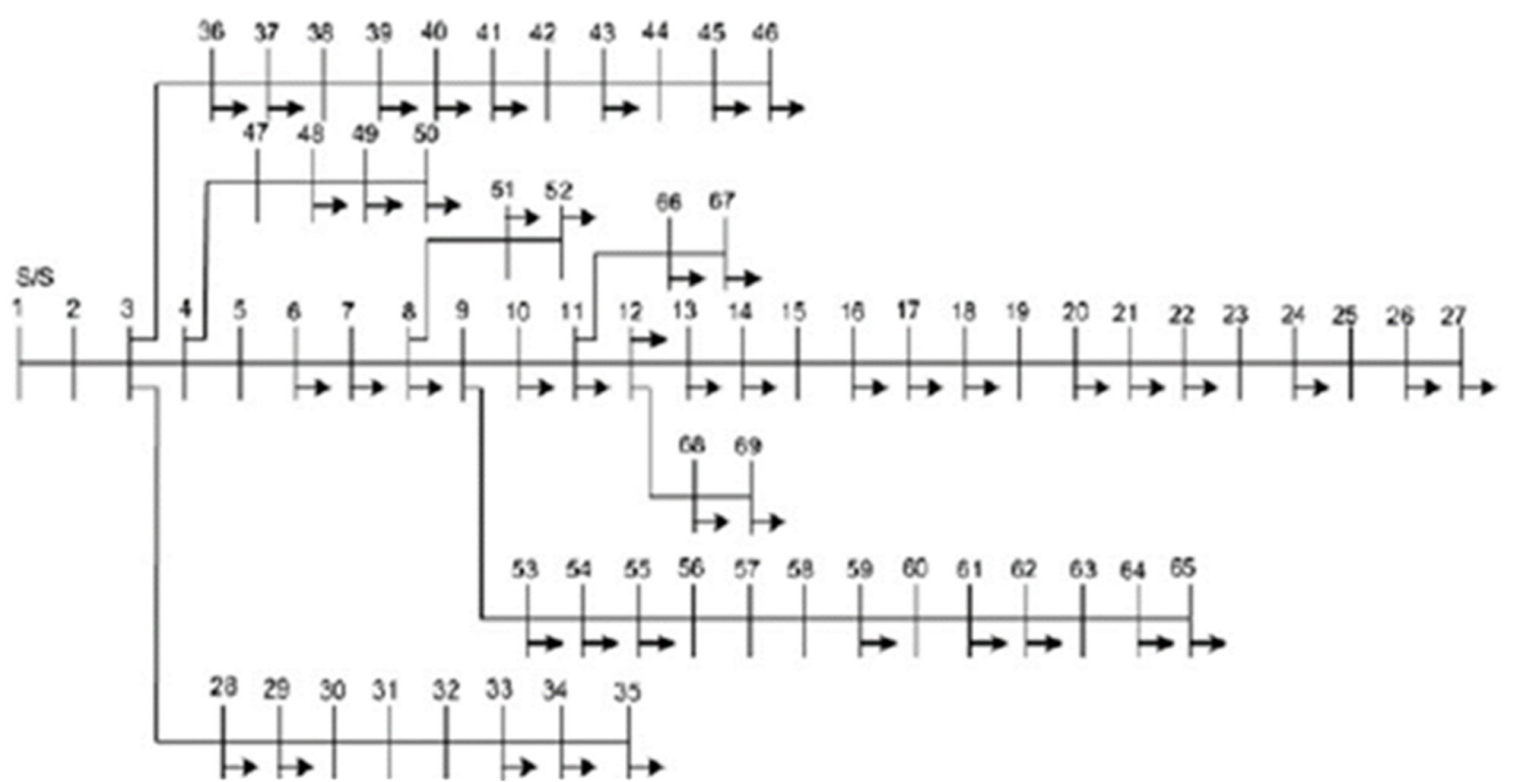
Comparative analysis of the AOASSA against the competitive optimization algorithms for the optimal allocation of (a) Single DG unit, (b) two DG units, (c) three DG units in the 33-bus RDN.

The standard 69-bus RDN has a balanced load configuration with an active load of 3.80 MW and the reactive load of 2.69 MVAr. A step-down transformer before node 1 reduces the voltage to 12.66 kV. The real power losses of 69-bus RDN without DG units were observed to be 225 kW. A standard 69-bus RDN is shown in Fig 10.

### Power loss minimization in 69-bus RDN

The decision variables for the 69-bus RDN are DG size and DG placement. The AOA outperforms the SSA and PSO in low-dimensional problems, whereas the SSAPSO, AOAPSO, and AOASSA demonstrate similar characteristics as presented in Table 9. Furthermore, the AOA results suggest that a 1795.1 kW DG should be placed at bus 61, the SSA results indicate that a 1734.9kW DG should be installed at bus 61, and the PSO results suggest that a DG of 1859.2 kW should be installed at bus 62 for the least losses. The hybrid SSAPSO, AOAPSO, and AOASSA reveal that the optimal size and position of one DG unit in a 69-bus RDN is 1872.7 kW at bus 61. In case 2, the AOA outcomes indicate DGs of 562.18 kW DG at bus 17 and 1775.6 kW at bus 61, SSA outcomes indicate that 546.1 kW at bus 17 and 1676.4 kW DG at bus 61, and PSO outcomes indicate that DG of 1723.1 kW and 842.08 KW should be placed at buses 61 and 66, respectively for the most minimized losses. On the other hand, the hybrid SSAPSO, AOAPSO and AOASSA reveal the best size and position of two DG units is 531.48 kW at bus 17 and 1781.4 kW at bus 61, respectively. Due to the simultaneous allocation of three DG units in case3, the DG allocation complexity increases as compared to cases 1 and 2. The AOA attains the DG sizes and locations that produced the least losses compared to SSA and PSO individually, as evident from Table 9. However, the power losses are still more significant than that of the proposed three hybrid combinations. AOASSA suggests DG sizes and locations which result in better performance than SSAPSO. In all three cases, the AOA outperforms the SSA and PSO due to the diversity of particles generated by multiplication and division operators in the exploration and subtraction and addition operators in the exploitation phase, which helped prevent local optima stagnation.

**Table 9:** Performance of the proposed AOASSA in 69-bus RDN with single and multiple DGs.

Case	Optimization Techniques	DG Size, kW (Bus location)	Power loss (kW)	PLR%
Case 1	AOA	1795.1 (61)	83.43	62.92
	SSA	1734.9(61)	83.89	62.72
	PSO	1859.2(62)	84.73	62.34
	SSAPSO	1872.7(61)	83.22	63.01
	AOAPSO	1872.7(61)	83.22	63.01
	AOASSA	1872.7(61)	83.22	63.01
Case 2	AOA	562.18 (17), 1775.6(61)	71.71	68.13
	SSA	546.1(17), 1676.4(61)	72.06	67.98
	PSO	1723.1(61), 842.08(66)	74.51	66.89
	SSAPSO	531.48(17), 1781.4(61)	71.67	68.14
	AOAPSO	531.48(17), 1781.4(61)	71.67	68.14
	AOASSA	531.48(17), 1781.4(61)	71.67	68.14
Case 3	AOA	358.84(18), 1703.8(61), 679.49(51)	70.58	68.63
	SSA	349.02(67), 1691.3(61), 416.37(27)	71.67	68.15
	PSO	1313.5(62), 518.79(17), 510.67(60)	72.32	67.86
	SSAPSO	399.78(12), 1748.8(61), 327.23(22)	69.70	69.02
	AOAPSO	1718.9(61), 526.84(11), 380.35(18)	69.43	69.14
	AOASSA	526.84(11), 1718.9(61), 380.35(18)	69.43	69.14

### Convergence quality analysis

**Fig 11:**
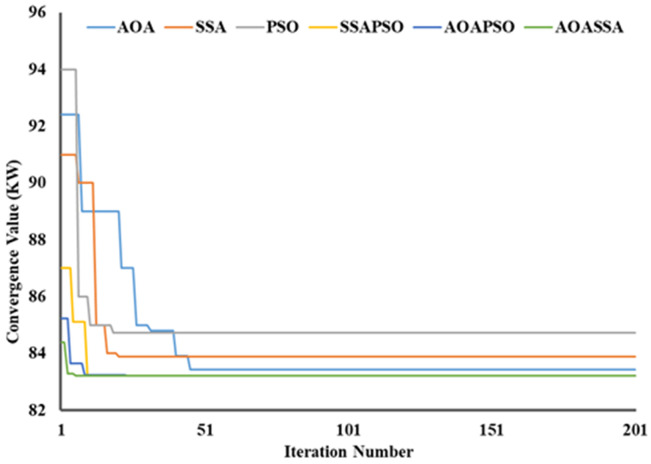
Convergence characteristics of contending optimization techniques for optimal allocation of single DG unit in the 69-bus RDN.

A high solution diversity (highly non-uniform) at the start of the search mechanism (exploration phase) and a lower diversity (almost uniform solution) at the end of the search mechanism (exploitation phase) is desired to obtain quality solutions. The proposed AOASSA running in parallel taxonomy produce quality solutions. The SSA has superior exploration capabilities than AOA and PSO when algorithms are compared individually and is evident from Fig 11. In contrast, the exploitation capabilities of AOA are superior to counterparts in case 1. In the first iteration, the AOA decreased power losses from 225 kW to 92.4 kW (58.93%), SSA reduced power losses to 91 kW (59.56%). PSO reduced power losses to 94 kW (58.22%), addressing the superiority of SSA in the exploratory phase (due to higher diversification produced by *c*_1_). In terms of exploitation capabilities, AOA converged to 83.43 kW (62.92%), SSA to 83.89 kW (62.72%), and PSO to 84.73 kW (62.34%), showing the exploitation potential of AOA owing to denser solutions provided by AOA at the end of the search process. The dominance of AOASSA against its counterparts (SSAPSO and AOAPSO) in exploration and exploitation is evident from Fig 11. The SSAPSO and AOAPSO have chosen a search space with poor exploration compared to AOASSA, as shown in Fig 11. The SSAPSO reduced the losses from 225 kW to 87 kW (61.33%), the AOAPSO reduced the losses to 85.22 kW (62.12%). The proposed AOASSA reduces the losses to 84.4 kW (62.49%) in the first iteration while dealing with the allocation of a single DG unit. The iterative parameter in SSA (*c*_1_) supports the exploration capabilities, while the iterative parameter in AOA supports the later exploitation phase of the search process.

**Fig 12:**
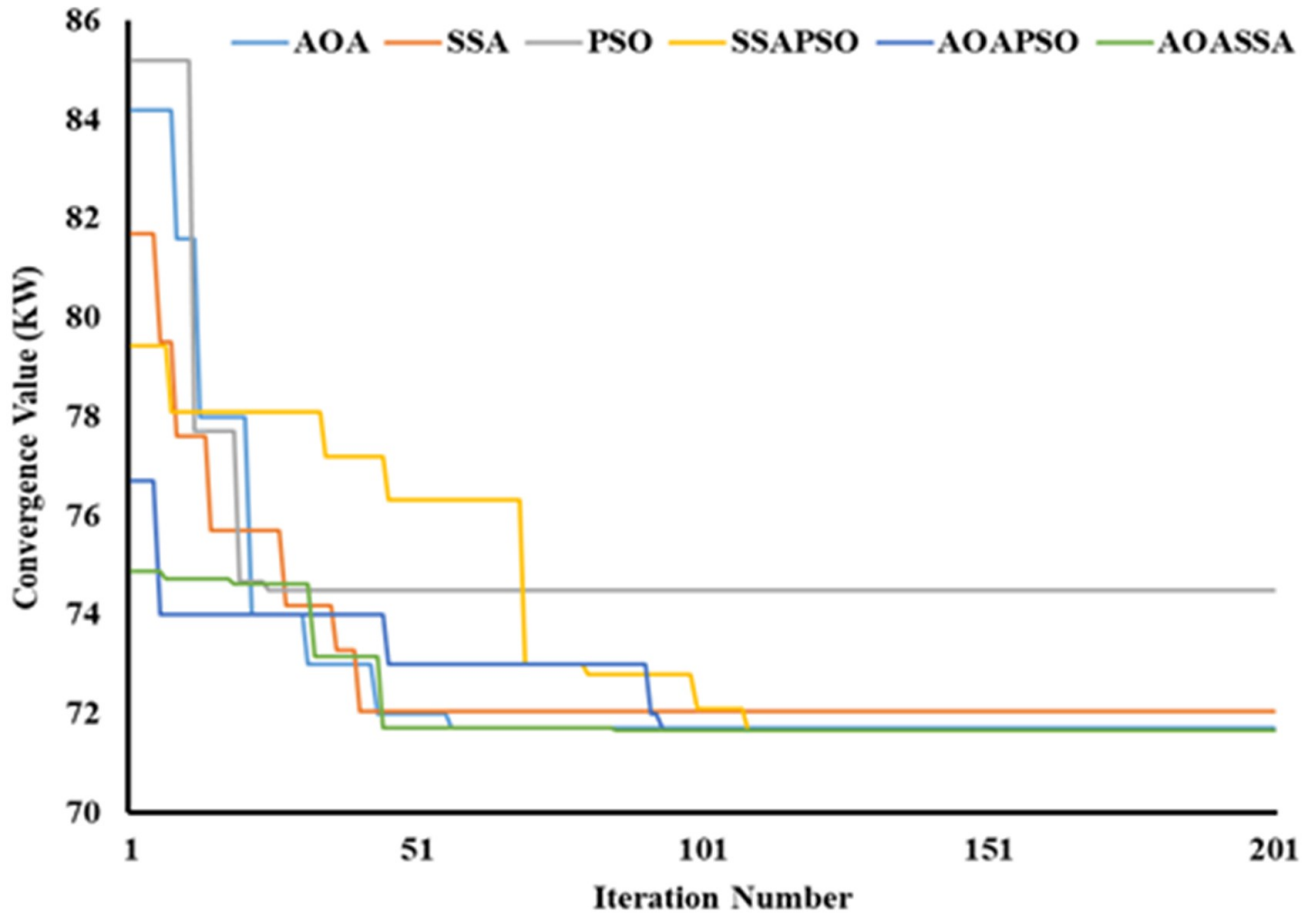
Convergence characteristics of contending optimization techniques for optimal allocation of two DG units in the 69-bus RDN.

Similarly, while dealing with the allocation of two DG units, the AOA decreases power loss from 225 kW to 84.2 kW (62.58%), SSA lowers power loss to 81.7 kW (63.69%). The PSO reduces power loss to 85.2 kW (62.13%), showing better exploration capabilities of SSA, as shown in Fig 12. Later in the exploitation phase, AOA takes the lead in the exploitation phase, picking an appropriate region with the least amount of particle diversification. As a result, the AOA converged to 71.71 kW (68.13%), the SSA converged to 72.06kW (67.97%), and the PSO converged to 74.5 kW (66.89%), showing the dominance in the exploitation phase of AOA. Thus, the proposed AOASSA outperformed in terms of exploration and exploitation capabilities. In the first iteration, the SSAPSO lowers losses from 225 kW to 79.42 kW (64.70%), the AOAPSO reduces losses to 76.7 kW (65.91%) and the proposed AOASSA decreases losses to 74.88 kW (66.72%) while dealing with the allocation of two DG units. Furthermore, the SSAPSO, AOAPSO, and AOASSA converges to the same power loss value during the exploitation phase. The results show that AOASSA has greater exploration and exploitation capabilities than its counterpart, as a parallel operated AOASSA provides better search inside the search space.

**Fig 13:**
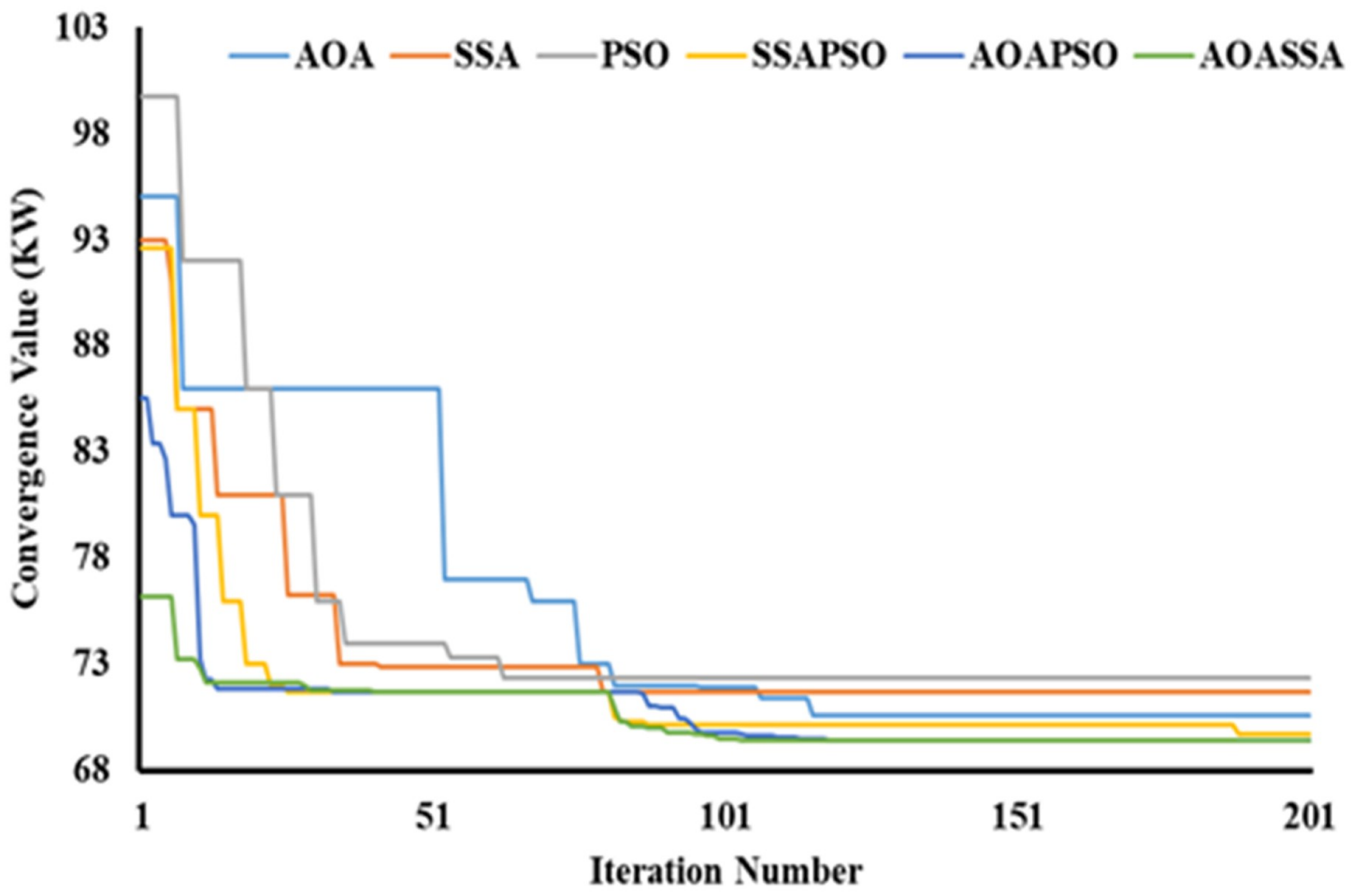
Convergence characteristics of contending optimization techniques for optimal allocation of three DG units in the 69-bus RDN.

In the third case, the AOA converges to 95 kW (57.78%), SSA converges to 93 kW (58.67%), and PSO converges to 99.7 kW (58.69%) in the first iteration, showing the exploratory capabilities of SSA at the start of the search phase. In terms of solution convergence, the AOA outperforms the SSA and PSO at the end of the search process, as shown in Fig 13. The AOA reduces the power loss to 70.58 kW (68.63%), SSA reduced the power loss to 71.67 kW (68.15%), and PSO reduces the power loss to 72.32kW (67.86%), highlighting the exploitation capabilities of AOA. Thus, the proposed AOASSA outperforms the hybrid algorithm in terms of exploration and exploitation capabilities. In the first iteration, the SSAPSO lowers losses from 225 kW to 92.56 kW (58.86%), the AOAPSO reduces losses to 85.49 kW (62%), and the proposed AOASSA decreases losses to 76.18 kW (66.14%) while dealing with the allocation of three DG units. It must be noted that the proposed AOASSA has superior exploitation capabilities as compared to SSAPSO, which stuck to local optima when three DG units are allocated. However, the exploitation capabilities of AOASSA are similar to AOAPSO as the power losses obtained by AOAPSO and AOASSA are the same at the end of 200 iterations. The proposed AOASSA combines the advantages of iterative parameter *c*_1_ in SSA during the exploration phase (resulting in considerable divergence at the start of the search process) MOP, subtraction, and addition operators at the end of the search process.

### Convergence speed analysis

Convergence speed is influenced directly by the diversity of particles in the search space and is quantified in terms of the number of iterations required to get the optimal solution. The algorithm is fast if the number of iterations needed to obtain the best solution is minimum. Convergence time is also affected by the complexity of the problem (in terms of network structure 69-bus structure is more complex than 33 bus network). In terms of DG allocation, increasing the number of DG units increases the complexity of the problem (due to increment in search space). The AOASSA help each other during exploration and exploitation, and the solutions quickly converge in search towards solution. In case1, AOA is slower than SSA and PSO as it takes 45 iterations to reach the best solution, SSA takes 20 iterations, and PSO takes 18 iterations. In hybrid algorithms, the AOASSA achieves the best DG size and position to produce the least power losses in 5 iterations, followed by the AOAPSO that reaches the optimal DG size and location to generate the least power losses in 24 iterations. SSAPSO requires 37 iterations to find the optimal DG size and position resulting in the minimum losses. In case 2, AOA takes 56 iterations to converge, SSA takes 40 iterations, and PSO takes 24 iterations to converge. AOASSA takes 85 iterations to reach the best DG sizes and sites in hybrid algorithms, followed by AOAPSO which takes 93 iterations and SSAPSO takes 108 iterations. Similarly, in case 3, AOA converges to the optimal solution in 115 iterations, SSA converges in 79 iterations, and PSO converges in 62 iterations. In the case of the hybrid algorithms, 111 iterations are required by the AOASSA to obtain optimal DG size and location, followed by the AOAPSO which takes 131 iterations and SSAPSO with 188 iterations to converge to minimum losses value. The AOASSA and AOAPSO converge to the same power loss value; however, the AOASSA responds faster than the AOAPSO due to solution diversity (better exploration and exploitation capabilities) and is evident from Table 10. As the number of DG allocation units increase, the number of iterations required to achieve the optimum solution also increase in similar manner. Furthermore, the iterations required to reach an optimal solution (in 69 bus network) are higher than to 33-bus network due to the complexity of the problem (higher search space). It is worthy to highlight that though the SSA and PSO are fast, yet they suffer from local optima stagnation and generate lower quality solutions than the AOA. The AOASSA outperforms in terms of convergence speed due to the superior exploration capabilities at the start of the search process and exploitation without the loss of diversity.

**Table 10:** Convergence speed of proposed algorithms with multiple DG allocation units.

Case	Techniques	Minimum iteration to reach global best
Case 1	AOA	45
	SSA	20
	PSO	18
	SSAPSO	37
	AOAPSO	24
	AOASSA	5
Case 2	AOA	56
	SSA	40
	PSO	24
	SSAPSO	108
	AOAPSO	93
	AOASSA	85
Case 3	AOA	115
	SSA	79
	PSO	62
	SSAPSO	188
	AOAPSO	131
	AOASSA	111

### Statistical analysis

The MHTs are stochastic in nature, and the mean, variance, and standard deviation are used to evaluate the performance superiority of algorithms. Similar to 33-bus RDN, the presented algorithms are executed 30 times to verify the robustness of solutions found in the case of the DG allocation problem. The AOA provides the lowest mean, variance, and standard deviations when MHTs are compared separately (AOA provides comparatively higher divergence at the start of the search process and denser solutions in the end). However, due to the iterative parameter, the inherent exploration abilities are lacking in MOP. As a result, a hybridization of AOA and SSA mitigates inherent exploratory limitations of AOA. The proposed AOASSA has a lower mean, variance, and standard deviation, as shown in Table 11, which are calculated based on the basis of best and worst values obtained in every execution. In addition, the proposed AOASSA has more robust exploration and exploitation mechanism supported by the pros of both SSA and AOA, resulting in substantially less dependent on the initial population. The proposed AOASSA is comparatively less dependent on the initial population generated and guarantees low mean, variance, and standard deviations due to iterative parameter *c*_1_ of SSA, followed by the better exploitation capabilities provided by the iterative MOP parameter of AOA. As a result, the proposed AOASSA has promising statistical characteristic compared to its counterparts, as shown in Table 11.

**Table 11:** Statistical superiority of AOASSA.

Case Number	Technique	Mean Power Loss (kW)	Standard Deviation	Variance
Case 1	AOA	86.54	3.76	14.14
	SSA	89.11	4.70	22.05
	PSO	89.33	5.19	26.97
	SSAPSO	84.53	1.75	3.08
	AOAPSO	84.03	1.57	2.45
	AOASSA	83.94	0.83	0.69
Case 2	AOA	74.91	3.87	14.98
	SSA	76.98	4.73	22.35
	PSO	79.49	5.86	34.35
	SSAPSO	74.30	2.15	4.60
	AOAPSO	74.04	1.95	3.79
	AOASSA	73.97	1.00	1.00
Case 3	AOA	74.38	4.15	17.21
	SSA	76.13	4.93	24.32
	PSO	80.18	7.27	52.90
	SSAPSO	73.74	2.16	4.68
	AOAPSO	72.23	2.10	4.40
	AOASSA	70.94	1.38	1.89

### Cost analysis

**Fig 14:**
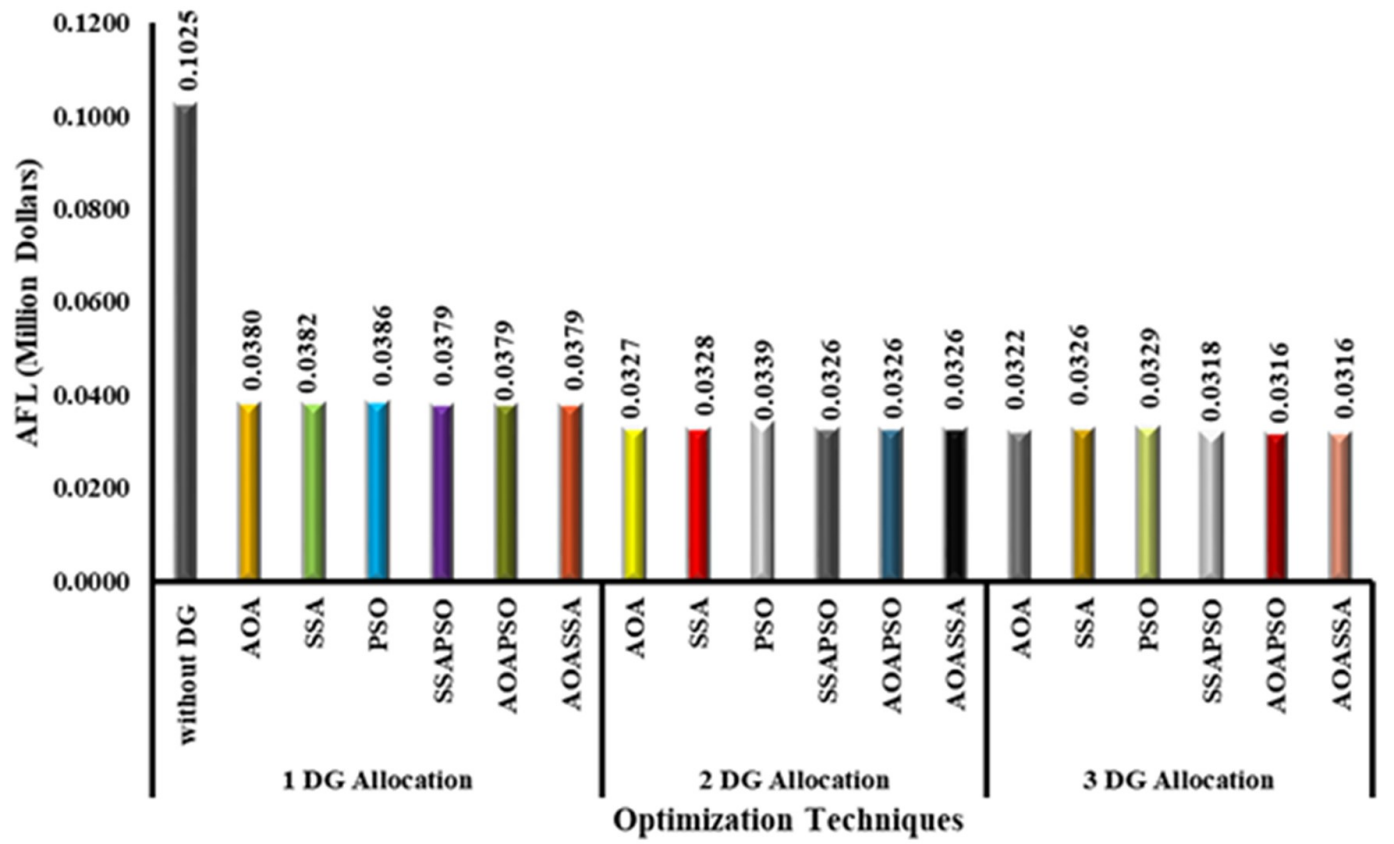
Annual financial losses with multiple DG units for different algorithms.

The primary goal of implementing the proposed AOASSA is to choose the best bus placement and size of DG which results in minimized losses. In case 1, the AOA, SSA, and PSO produces the optimal sizes and positions reducing power losses by 62.92%, 62.72%, and 62.34%, respectively, lowering the AFL from 0.1025 million dollars to 0.0380, 0.0382, and 0.0386 million dollars, respectively. For SSAPSO, AOAPSO, and AOASSA, the optimal allocation of one DG unit reduced power losses by 62.01%, lowering the proportionate cost of losses. With the optimal allocation of one DG unit, the cost of losses decreased from 0.1025 million dollars to 0.0379 million dollars, regardless of the hybrid optimization technique used as shown in Fig 14. In case 2, the optimal sizes and positions of two DG units produced by AOA, SSA, and PSO reduced power losses by 68.13%, 67.98%, and 66.89%, respectively. The AOA, SSA, and PSO reduce the AFL from 0.1025 million dollars to 0.0327, 0.0328, and 0.0329 million dollars. Furthermore, the optimal allocation of two DG units reduced losses by 68.14%, lowering financial losses with the same ratio. Regardless of the hybrid optimization technique used (SSAPSO, AOAPSO, or AOASSA), the cost of losses decreased from 0.1025 million dollars to 0.0326 million dollars in proportion to PLR. In case 3, the optimal sizes and positions of three DG units produced by AOA, SSA, and PSO reduced power losses by 68.63% (from 0.1025 million dollars to 0.0322), 68.15% (from 0.1025 million dollars to 0.0326), and 67.86% (from 0.1025 million dollars to 0.0329), respectively. In case 3, when three DG units were optimally allocated, power losses dropped to 69.02% for SSAPSO and 65.14% % for AOAPSO and AOASSA. As a result, the proportional cost of losses for SSAPSO has been decreased from 0.1025 million dollars to 0.0318 million dollars. In comparison, the cost of losses for AOAPSO and AOASSA has been reduced from 0.1025 million dollars to 0.0316 million dollars. The cost of losses for each of the three cases are shown in Fig 14.

### Benchmarking on 69-bus RDN

**Fig 15:**
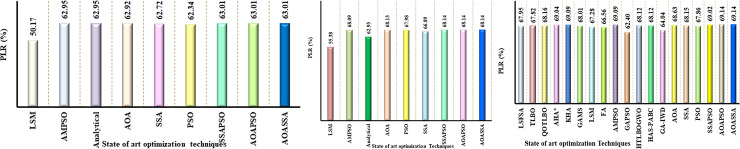
Comparative analysis of the AOASSA against the competitive optimization algorithms for the optimal allocation of (a) Single DG unit, (b) Two DG units, (c) Three DG units in the 69-bus RDN.

The optimal sizes and positions of DGs for some recent MHTs are listed, the corresponding power loss reductions are presented in Table 12. A summary of PLR obtained with multiple DG units from literature has been presented in Fig 15. The proposed AOASSA provides superior results in terms of PLR, as shown in Fig 15A, 15B and 15C.

**Table 12:** Optimal capacities and positions of DGs in the 69-bus RDN for the proposed and benchmarked algorithms.

Optimization Techniques	1 DG		2 DGs		3 DGs	
	DG Size (kW), (@Bus)	PLR (%)	DG Size (kW), (@Bus)	PLR (%)	DG Size (kW), (@Bus)	PLR (%)
LSF-SA [51]	-	-	-	-	420.4 (18) 1331.1 (60) 429.8 (65)	67.95
TLBO [34]	-	-	-	-	591.9 (15) 818.8 (61) 900.3 (63)	67.82
QO-TLBO [34]	-	-	-	-	533.4 (18) 1198.6 (61) 567.2 (63)	68.16
AHA [64]	-		-	-	471 (12) 312 (21) 1689(61)	69.04
KHA [35]	-	-	-	-	496.2(12) 311.3 (22) 1735.4 (61)	69.09
LSM [66]	1436.3 (65)	50.17	1379.1 (65) 446.1 (27)	55.38	196.6 (65) 416.8 (27) 1602.6 (61)	67.28
Analytical [31]	1800 (61)	62.95		-	-	62.95
GAMS [7]	-	-	-	-	813.1 (12) 1444.7 (61) 289.6 (64)	68.01
FA [65]	-	-	-	-	295.4 (27) 447.6 (65) 1345.1 (61)	66.56
AM-PSO [48]	1810 (61)	62.95	520 (17) 1720 (61)	68.09	510 (11) 380 (17) 1670 (61)	69.09
Hybrid Teaching–Learning Based Optimization-Grey Wolf Optimizer HTLBOGWO [67]	-	-	-	-	533 (18) 1000 (61) 773 (62)	68.12
GAPSO [52]	-	-	-	-	910.5 (21) 1192.6 (61) 884.9 (63)	62.40
HSAPABC [55]	-	-	-	-	530 (18) 1000 (61) 7730 (62)	68.12
GA-IWD [54]	-	-	-	-	911.5 (20) 1392.6 (61) 805.9 (64)	64.04
AOA	1795.1 (61)	62.92	562.18 (17) 1775.6(61)	68.13	358.84 (18) 1703.8 (61) 679.49 (51)	68.63
SSA	1734.9 (61)	62.72	546.1(17) 1676.4(61)	67.98	349.02 (67) 1691.3 (61) 416.37 (27)	68.15
PSO	1.8592 (62)	62.34	1723.1(61) 842.08(66)	66.89	1313.5 (62) 518.79 (17) 510.67 (60)	67.86
SSAPSO	1872.7 (61)	63.01	571.58(17) 1768.2(61)	68.14	399.78 (12) 1748.8 (61) 327.23 (22)	69.02
AOAPSO	1872.7 (61)	63.01	531.48(17) 1781.4(61)	68.14	1718.9 (61) 526.84 (11) 380.35 (18)	69.14
**AOASSA (Proposed)**	**1872.7 (61)**	**63.01**	**531.48(17) 1781.4(61)**	**68.14**	**526.84 (11) 1718.9 (61) 3803.5 (18)**	**69.14**

The statistic illustrates the PLR achieved by recent state of the art algorithms involving multiple DG units. Initially, the findings are compared to low-dimensional benchmarks (1 DG unit allocation. It can be analyzed from Fig 15 that the power loss reduction obtained by LSM [66] is 50.17%, Analytical [31] is 62.95%, AMPSO [48] is 62.95%, AOA is 62.92%, 62.72%, PSO is 62.34%, SSAPSO is 63.01, AOASSA is 63.01 and AOASSA is 63.01%. In case2, the PLR obtained by LSM [66] is 55.38%, AMPSO [48] is 68.09%, AOA is 68.13, SSA is 67.98, PSO is 66.89%, SSAPSO is 68.14%, AOAPSO is 68.14%, and AOASSA is 68.14%. In case 3, the PLR obtained by LSFSA [51] is 67.95%, TLBO [34] is 67.82%, QOTLBO [34] is 68.16%, AHA [64] is 69.0369%, KHA [35] is 69.09%, LSM[66] is 67.28%, GAMS [7] is 68.01%, FA [65] is 66.56%, AMPSO [48] is 69.09%, Hybrid Teaching Learning Based Optimization-Grey Wolf Optimizer (TLBOGWO) [67] is 68.12%, GAPSO [52] is 62.40%, HSAPABC [55] is 68.12%, GAIWD [54] is 64.04%, AOA is 68.63%, SSA is 68.15%, PSO is 67.86%, SSAPSO is 69.02%, AOAPSO is 69.14%, and AOASSA is 69.14%. The exceptional hybrid combination of AOASSA supporting each other during the exploration (SSA supporting AOA) and exploitation phase (AOA supporting SSA) is the reason AOASSA outperforms compared to other algorithms.

### Interpretations based on obtained results

This section provides an argument-based discussion interpreting the results obtained in detail.

#### Interpretations on power loss reduction

The optimized sizes and locations obtained from AOA, SSA and PSO resulted in a slight difference in terms of PLR. The arithmetic optimization algorithm provides the highest diversity of particles due to the division and multiplication operators. The exploitation mechanism is followed by lower divergence due to addition and subtraction operators. However, the iterative parameter MOP lacks the exploration capabilities. Contrary, the SSA provides a sorting mechanism at the start of search process and is followed by updating leader and follower positions. However, the solutions have higher divergence in the exploitation phase, resulting in comparatively higher power loss compared to AOA. At the same time, PSO results in the highest power losses compared to other standard algorithms due to the linear search mechanism provided by inertia weight. The performance of hybrid algorithms in terms of power loss reduction is same when dimensions of the problem are low (with one and two DG unit(s) allocation). However, with increasing dimensions (allocation of 3 DG units), the SSAPSO has comparatively higher power losses due to the absence of mathematical operators (division, multiplication, subtraction, and addition) which provides significantly higher divergence at the start of search mechanism and lower divergence at the end of search mechanism. AOASSA and AOAPSO have equal performance due to the presence of mathematical operators and supporting iterative parameters at different stages of the search process.

#### Interpretations on solution quality

The solution quality has been observed in terms of exploration and exploitation capabilities amid the algorithms used. The SSA has the greatest exploration capabilities due to its iterative parameter *c*_*1*_ but lacks the exploitation capabilities (which can be observed prominently with the allocation of 3-DG units). On the contrary, the AOA has great exploitation capabilities but lacks in the exploration phase. At the same time, PSO has the least diversity of particles in the exploration phase and comparatively higher divergence in the exploitation phase (due to fast converging inertia weight) that led to higher power losses (most prominent while dealing with 3 DG allocation). However, among hybrid algorithms, the performance of AOASSA is found to be superior amid the contending techniques due to the iterative parameters MOP and *c*_*1*_. On the contrary, SSAPSO lacks the mathematical operators, resulting in weak exploration and exploitation search.

#### Interpretations on convergence speed

The convergence speed among the standard versions of AOA, SSA, PSO and their possible combinations have been observed. The algorithm that reaches the global best with the least number of iterations is considered the fastest algorithm. However, it must be noted that the global best values (power loss value) obtained are not the same (especially when dimensions of the problem are high). AOA is comparatively slower as compared to other standard versions of SSA and PSO since PSO has linearly decreasing inertia weight, which has a faster decline than contending iterative parameters (c_*1*_ and MOP). However, among the hybrid algorithms, AOASSA converges fast (especially with higher dimension problems) due to the diversity of the particles provided by the iterative changing parameters c_1_ and MOP.

#### Interpretations on statistical analysis

The considered algorithms are stochastic which produce randomly generated set of DG size and locations. The statistical analysis is carried out to test the robustness of standard versions of AOA,SSA,PSO and their possible hybrid combinations. The algorithms having dependencies on initial solutions are usually less robust and have higher variations in individual executions. The AOA algorithm has an excellent search mechanism that produces quality solutions due to the presence of mathematical operators but lacks better exploration. The mentioned weakness is mitigated with the iterative parameter of SSA. PSO has linearly decreasing inertia weight providing fast convergence but does not guarantee a quality solution. The parallel operated AOASSA tends to provide higher diversion of particles in exploration and lesser divergence of particles in exploitation phase, which provides a greater chance to converge to optimal solution for individual execution and converge to minimal power losses.

#### Interpretations on Annual Financial losses (AFL)

The appropriate placement and size of these DGs can help to cut down on energy losses and the annual monetary losses. The proposed study considers AOA, SSA, PSO and the possible combinations for analyzing the AFL. The AFL are purely dependent on system power losses which in turn depends on the sizes and locations of DG units in the distribution network. Among the set of algorithms, the least power losses are produced by AOASSA and AOAPSO, and hence least financial losses are achieved with these hybrid combinations.

#### Interpretations on benchmarking

Although a variety of analytical, metaheuristic and hybrid algorithms are presented in the literature but have some methodological limitations. The solutions obtained from various algorithms and the associated control variables (sizes and locations) are presented in Tables 8 and 12 (for 33 and 69 bus networks) respectively. It is evident that the proposed parallel hybrid combination of AOASSA has the highest power loss reduction compared to the presented and contending algorithms available in the literature.

## Conclusion and future roadmaps

From the brief review of literature, it can be concluded that the hybrid techniques are more capable for optimal allocation of DG units. However, the existing hybrid techniques have some taxonomical limitations. The inherent limitations in the hybrid techniques lead to non-optimal solutions of DG sizes and locations, leading to increased power losses in RDNs. To address such taxonomical limitations, the article proposes parallel operated hybrid AOASSA for minimizing active power losses with optimal allocation of DG units in AC distribution networks. The proposed hybrid mechanism initiates by running AOA and SSA independently, compares the power loss reduction and replace the control variables with the dominant solution in each run. The numerical results confirms that the proposed parallel operated AOASSA shows superior results. Among the parallel operated hybrid optimizers, the PLR and associated AFL obtained with AOASSA, and AOAPSO is highest (i.e., 65.51% and 69.14% for 33-bus and 69-bus systems, respectively). Furthermore, the proposed AOASSA is more robust and converge faster towards best solution compared to contending algorithms as the final solutions do not depend on the initial solution in each run. The proposed parallel operated AOASSA optimizer is an efficient optimization technique to determine the optimal sizes and location of DG units and is benchmarked against recent optimization algorithms with the help of 3 cases. In future, the parallel hybrid AOASSA can be applied to control problems. The upcoming researchers can vary the default parameters of algorithms in order to observe the improvements in PLR. In addition, the study is focused on minimizing the power losses, while the study may be extended to a multi-objective function.

## Supporting information

S1 Data (RAR)

S1 File (IN)

S2 File (IN)
