# Correction: Parallel operated hybrid Arithmetic-Salp swarm optimizer for optimal allocation of multiple distributed generation units in distribution networks

**DOI:** 10.1371/journal.pone.0294704

**Published:** 2023-11-15

**Authors:** Zeeshan Memon Anjum, Dalila Mat Said, Mohammad Yusri Hassan, Zohaib Hussain Leghari, Gul Sahar

The first author’s name is spelled incorrectly. The correct name is: Zeeshan Anjum Memon.

The correct citation is: Memon ZA, Said DM, Hassan MY, Leghari ZH, Sahar G. (2022) Parallel operated hybrid Arithmetic-Salp swarm optimizer for optimal allocation of multiple distributed generation units in distribution networks. PloS ONE 17(4): e0264958. https://doi.org/10.1371/journal.pone.0264958
